# Synthesis of 2-Deoxyglycosides with Exclusive β-Configuration Using 2-SAc Glycosyl Bromide Donors

**DOI:** 10.3390/molecules30010185

**Published:** 2025-01-05

**Authors:** Yang-Fan Guo, Tian-Tian Xu, Guo-Hui Zhang, Hai Dong

**Affiliations:** Key Laboratory of Material Chemistry for Energy Conversion and Storage, Hubei Key Laboratory of Bioinorganic Chemistry and Materia Medica, School of Chemistry & Chemical Engineering, Huazhong University of Science & Technology, Ministry of Education, Luoyu Road 1037, Wuhan 430074, China

**Keywords:** 2-deoxyglycoside, β-configuration, glycosylation, Koenigs–Knorr condition, desulfurization

## Abstract

In this study, we developed an indirect method for the synthesis of 2-deoxyglycosides with an exclusive β-configuration using glucosyl and galactosyl bromide donors with 2-thioacetyl (SAc) groups. The 2-SAc glucosyl and galactosyl bromide donors were easily obtained through the treatment of 1-OAc, 2-SAc glucose and galactose with HBr-CH_3_COOH solution, respectively. The glycosylation of such donors with acceptors under an improved Koenigs–Knorr condition resulted in glycosylation products with an exclusive β-configuration in excellent yields. The synthetic approach of 2-SAc glycosyl donors using glycals as the starting materials was also investigated. Based on these studies, the synthetic method of using 2-deoxyglycosides with an exclusive β-configuration through desulfurization will have more practical applications.

## 1. Introduction

**Scheme 1:**
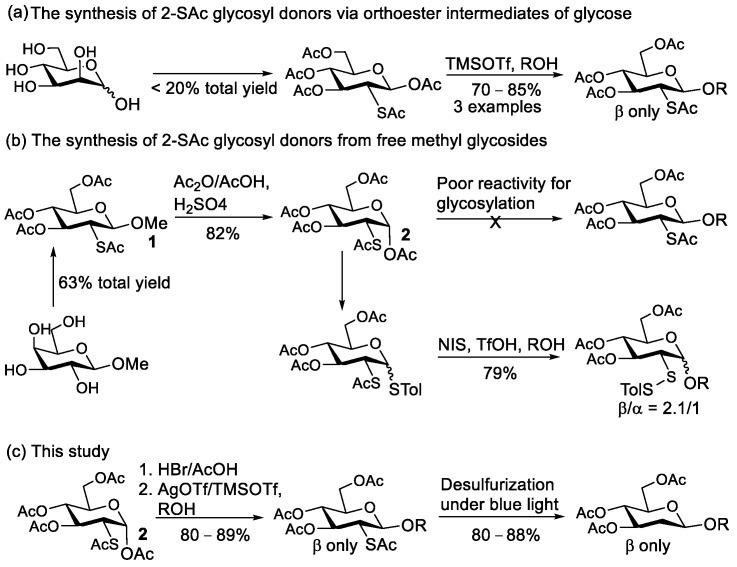
Strategies to obtain 2-SAc glycosyl donors for the synthesis of 2-deoxyglycosides [17,19,21].

In the synthesis of 2-deoxyglycosides, which are important components of biologically active oligosaccharides and natural products, the control of anomeric stereochemistry is a big challenge due to the lack of C-2 oxygen [1,2,3,4,5,6,7,8,9,10,11,12,13,14]. There are two common ways, namely the direct or indirect strategy, that can stereocontrol their synthesis. The direct strategy allows for the direct generation of 2-deoxy products via coupling reactions between activated glycosyl donors and nucleophilic acceptors [4,5,6,7,8,9,10]. However, it is usually more favorable to afford 2-deoxy α-glycosides due to the anomeric effect [4,5,6,7,8]. To obtain 2-deoxyglycosides with an exclusive β-configuration, an indirect strategy is usually necessary [11,12,13,14,15,16,17]. The indirect approach involves the glycosylation of glycosyl donors with a temporary directing group at the C-2 position and the removal of the temporary directing group after glycosylation. Knapp first introduced thioacetate (SAc) at C-2 of a glycosyl donor (1-OAc, 2-SAc glycoses) and used Raney-nickel-catalyzed desulfurization to synthesize 2-deoxyglycosides with an exclusive β-configuration using an indirect strategy [17]. We later developed efficient light-induced desulfurization methods and further applied desulfurization and 1-OAc, 2-SAc donors for the synthesis of 2-deoxyglycosides with an exclusive α/β-configuration [18,19,20,21,22]. Apparently, effective access to 2-SAc glycosyl donors is the key to making the indirect strategy of using SAc as a temporary directing group more practical. The methods for the synthesis of 1-OAc, 2-SAc glycosyl donors via orthoester intermediates of glycose (Scheme 1a) are very inefficient (total yields < 20%) [17,19]. Therefore, we first developed methods for the efficient synthesis of 1-OAc, 2-SAc donors (manno-, gluco-, galacto-, and talo-type α-glycosyl donors, with a total of 43–58% yields starting from free methyl glycosides) and then tested these donors in glycosylation [21]. The α-mannosyl and α-talosyl donors exhibited high reactivity in the test, whereas the α-glucosyl and α-galactosyl donors exhibited poor reactivity, leading to the failure of the synthesis of 2-deoxyglycosides with an exclusive β-configuration (Scheme 1b). This is because glycoside donors with a 1,2 *cis*-configuration usually exhibit low reactivity in glycosylation [23,24,25]. We then converted 1-OAc, 2-SAc α-glucosyl and α-galactosyl donors to 1-STol, 2-SAc glycosyl donors to improve the reactivity of the donors [21]. This attempt to obtain glycosylation products with an exclusive β-configuration remained unsuccessful due to the glycosylation products with α/β-isomers caused by the labile thioacetate group under the glycosylation conditions (Scheme 1b). It is well known that Koenigs–Knorr glycosylation usually involves the activation of glycosyl halide donors through the coordination of halides to silver salts, while glycosyl bromide donors can be obtained by the bromination of 1-OAc sugars [26,27,28,29,30]. We speculated that 2-SAc should be tolerated during Koenigs–Knorr glycosylation and that the glycosylation of 2-SAc bromide donors (gluco- and galacto-type) with acceptors should readily yield glycosylation products with an exclusive β-configuration. This is addressed in this study (Scheme 1c). Our results demonstrate that 2-deoxyglycosides with an exclusive β-configuration could be efficiently synthesized by this approach.

## 2. Results

We can easily synthesize 2-SAc glucosyl bromide donor through the treatment of 1,3,4,6-tetra-*O*-acetyl-2-*S*-acetyl-α-glucose 2 [21] with two equiv. of hydrobromic acid solution (HBr-CH_3_COOH) in CH_2_Cl_2_ at 0 °C. The crude donor product was obtained after extraction with CH_2_Cl_2_ and washing with saturated NaHCO_3_ and concentration, and it was directly tested in a glycosylation reaction with an acceptor **a** (Table 1). Demchenko’s group improved the traditional Koenigs–Knorr glycosylation method, where the addition of catalytic amounts of Lewis acid could greatly increase the reaction rate [26]. They optimized the glycosylation reaction conditions using 0.5 equiv. of Ag_2_O and 0.4 equiv. of TfOH in one study [30]. Thus, with these reaction conditions, glycosylation product **2a** with an exclusive β-configuration was isolated in a 65% yield after a 30 min reaction (entry 1). We then increased the amounts of Ag_2_O to 1.0 equiv. and the yield of **2a** increased to 78% in this case (entry 2). An acetyl migration product (obtained from the SAc group migrating to the 6-OH of **a**) [21] could be isolated from these reactions. Thioacetyl migration is likely due to the basicity of Ag_2_O and the labile SAc group even under weak basic conditions [20,31,32,33]. Based on this, 2.0 equiv. of AgOTf was used instead of 1.0 equiv. of Ag_2_O in a further test (entry 3). It can be seen that the yield of **2a** was increased to 88% after only 10 min of reaction due to the inhibition of acetyl migration. As the amounts of AgOTf were decreased to 1.0 equiv., the yield of **2a** was decreased to 75% even after 60 min of reaction (entry 4). We then increased the amounts of AgOTf to 1.2 equiv., by which the yield of **2a** was increased to 85% after 10 min of reaction (entry 5). We subsequently optimized the amount of Lewis acid catalyst. It can be seen that **2a** was still isolated in an excellent yield (87%) even as the amounts of TfOH were decreased to 0.1 equiv. from 0.4 equiv. (entry 6). When 0.1 equiv. of TMSOTf was used instead of TfOH, **2a** was isolated in an 89% yield (entry 7). Without the use of TfOH or TMSOTf, the reactivity of this glycosylation was drastically reduced, leading to a prolonged reaction time (6 h) and a reduced yield (64%, entry 8). These results indicate that it was feasible to obtain glycosylation products with an exclusive β-configuration by using 2-SAc glycosyl bromide donors.

**Figure 1:**
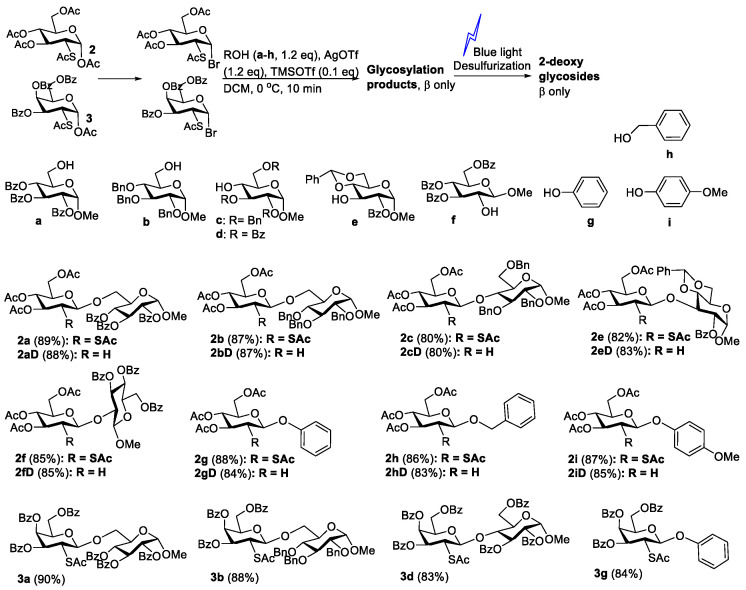
Glycosylation of 2-SAc glycosyl bromide donors with various acceptors and subsequent preparation of 2-deoxyglycosides with exclusive β-configuration.

With the optimized glycosylation conditions (1.2 equiv. of AgOTf and 0.1 equiv of TMSOTf) in hand, we further tested the glycosylation of 2-SAc glycosyl bromide donors with various acceptors (Figure 1). The glycosylation of the 2-SAc glucosyl bromide donor with acceptors **a**, **b**, **c**, **e**, **f**, **g**, **h**, and **i** led to products **2a**, **2b**, **2c**, **2e**, **2f**, **2g**, **2h**, and **2i** (β-configuration only) in 89%, 87%, 80%, 82%, 85%, 88%, 86%, and 87% yields, respectively. After that, the 2-deoxyglucosides **2aD**, **2bD**, **2cD**, **2eD**, **2fD**, **2gD**, **2hD**, and **2iD** (with an exclusive β-configuration) were obtained by the desulfurization of these glycosylation products under blue light [22] in 88%, 87%, 80%, 83%, 85%, 84%, 83%, and 85% yields, respectively. The crude 2-SAc galactosyl bromide donor was prepared by the treatment of 1-*O*-acetyl-3,4,6-tri-*O*-benzoyl-2-*S*-acetyl-α-glucose **3** [21] with two equiv. of HBr-CH_3_COOH solution in CH_2_Cl_2_ at 0 °C. The glycosylation of this donor with acceptors **a**, **b**, **d**, and **g** led to products **3a**, **3b**, **3d**, and **3g** (β-configuration only) in 90%, 88%, 83%, and 84% yields, respectively. We did not further validate the desulfurization of **3a**, **3b**, **3d**, and **3g** since the blue light-induced desulfurization proved to be a very effective method [22].

**Scheme 2:**
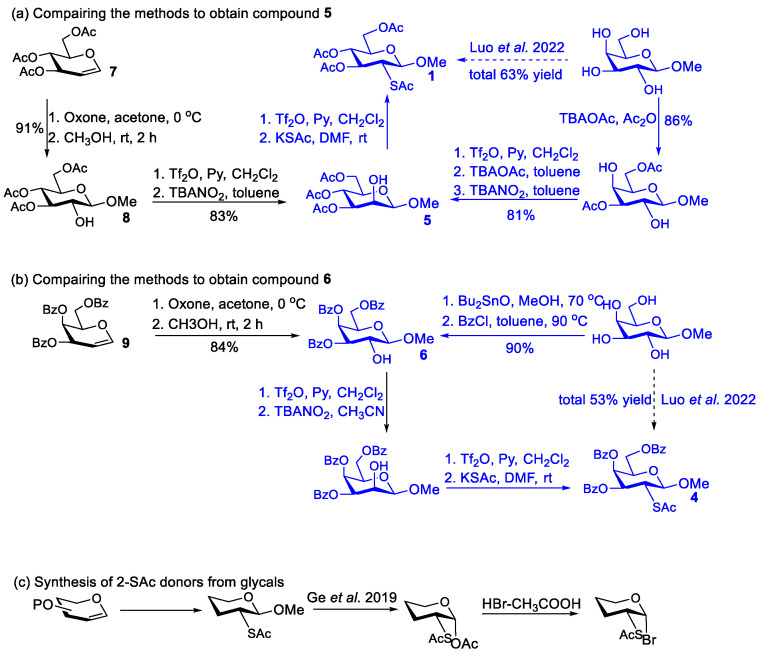
Synthesis of 2-SAc donors from glycals [19,21].

In our previous studies, 1-OAc, 2-SAc α-glycosyl donors **2** and **3** were synthesized from methyl 2-SAc β-D-glycosides **1** and **4** [21], which were synthesized starting from the unprotected methyl β-D-galactoside by a combination of selective protection operations and inversion operations [34,35,36,37] (Scheme 2a,b). We recently noticed that 2-OH glycosides could be easily synthesized from the corresponding per-protected glycals by means of 1,2-anhydro intermediates prepared by the oxidation of these glycals [38,39,40]. Therefore, we envisaged obtaining the key intermediate products (2-OH glycosides) **5** and **6** starting from glycals (Scheme 2a,b). The oxidation of 3,4,6-tri-O-acetyl glucal **7** with oxone in acetone afforded its corresponding 1,2-anhydro glucose product, and then the crude product was directly reacted with methanol (as a solvent also) at rt for 2 h to yield methyl 3,4,6-tri-*O*-acetyl-β-glucoside **8** in a 91% yield. The inversion of **8** by TBANO_2_ afforded **5** in an 83% yield. Similarly, the oxidation of 3,4,6-tri-*O*-benzoyl galactal **9** with oxone in acetone afforded its corresponding 1,2-anhydro galactose product, and then the crude product was directly reacted with methanol at rt for 2 h to yield methyl 3,4,6-tri-*O*-benzoyl-β-galactoside **6** in an 84% yield. Obviously, obtaining **6** using this method avoided the use of highly toxic organotin reagents (Scheme 2b). These results indicate that a variety of 2-SAc glycoside donors could be efficiently prepared starting from their corresponding glycals (Scheme 2c).

## 3. Materials and Methods

General methods. All chemicals were purchased as reagent grade and used without further purification. The solvents were purified before use. CH_3_CN was distilled over CaH_2_. Chemical reactions were monitored by thin-layer chromatography using precoated silica gel 60 (0.25 mm thickness) plates. Flash column chromatography was performed on silica gel 60 (SDS 0.040–0.063 mm). Spots were visualized by UV light (254 nm) and charred with a solution of H_2_SO_4_ in ethanol. ^1^H NMR and ^13^C NMR spectra were recorded at 298 K in CDCl_3_ or CD_3_OD using the residual signals from CDCl_3_ (^1^H: δ = 7.26 ppm) and CD_3_OD (^1^H: δ = 3.31 ppm) as internal standard.

General procedure A for the glycosylation of 2-SAc donors: 1-OAc-2SAc-glycose (0.12 mmol) in DCM (1 mL) was added to hydrobromic acid (33 wt.% in acetic acid) (1 mL) at 0 °C, and the mixture was stirred at 0 °C for 2 h. The resulting mixture was diluted with dichloromethane and washed with ice water, saturated aqueous NaHCO_3_, and brine. The collected organic phase was dried with MgSO_4_ and then concentrated under reduced pressure. After the mixture of the crude product, an acceptor (0.14 mmol), and 4 Å molecular sieves in dry CH_2_Cl_2_ (1.2 mL) was stirred at 0 °C under argon for 30 min, AgOTf (1.2 equiv) and TMSOTf (0.1 equiv) were added. Then, the reaction proceeded at 0 °C for 10 min, and the resulting mixture was quenched with TEA, filtered, concentrated, and chromatographed on a silica gel column (EtOAc/petroleum ether) to afford the pure product.

General procedure B for desulfurization under blue light: A substrate (50 mg) was allowed to react with hydrated hydrazine (1.8 equiv per thiol group) in DMF (1 mL) at room temperature for 2–5 min and monitored with TLC. TCEP·HCl (2.5 equiv), Ru(bpy)_3_Cl_2_ (0.02 equiv), and a molecular sieve were added to the reaction and stirred for a few mins. At last, the reaction was exposed to blue light (12 W, 460–470 nm) at rt for 10 h. The reaction mixture was concentrated in vacuo and directly purified by flash column chromatography (EtOAc/Petroleum ether = 1:4) to afford the pure product.

General procedure C for the oxidation of glycals by oxone: To a cooled (0 °C) solution of a protected glycal (0.36 mmol) in DCM (2 mL), acetone (0.2 mL) and saturated aqueous NaHCO_3_ (3.4 mL) were added. The mixture was stirred vigorously, and a solution of oxone (450 mg) in H_2_O (6 mL) was added dropwise over 15 min. The mixture was stirred vigorously at 0 °C for 30 min and then stirred at rt until TLC indicated the consumption of the starting material. The organic phase was separated, and the aqueous phase was extracted with DCM (2 × 10 mL). The combined organic phase was dried (MgSO_4_) and concentrated in vacuo to obtain the crude 1,2-anhydro sugar.

Compounds **1**, **2**, **3**, and **4** were synthesized in light of the reported literature [21].

Methyl 2,3,4-tri-*O*-benzoyl-6-*O*-(2-*S*-acetyl-3,4,6-tri-*O*-acetyl-2-thio-*β*-D-glucopyranoside)-*α*-D-glucopyranoside **2a** was synthesized by the use of **2** (50 mg, 0.12 mmol), methyl 2,3,4-tri-*O*-benzoyl-*α*-D-glucopyranoside (75 mg, 0.15 mmol), AgOTf (35 mg, 0.14 mmol), and TMSOTf (1.8 uL, 0.01 mmol) through general procedure A. The crude product was purified by flash column chromatography on a silica gel column (EtOAc/petroleum ether = 1:6) to afford **2a** as a colorless oil (93.4 mg, 0.109 mmol, 89% yield). Rf = 0.3 (ethyl acetate/petroleum ether 1:4); ^1^H NMR (400 MHz, CDCl_3_) δ 8.05–7.85 (m, 7H), 7.59–7.31 (m, 8H), 6.25 (t, *J* = 9.6 Hz, 1H), 6.14 (t, *J* = 9.5 Hz, 1H), 5.50 (dt, *J* = 16.6, 9.8 Hz, 1H), 5.33–5.20 (m, 3H), 5.05 (dd, *J* = 10.1, 9.0 Hz, 1H), 4.72 (d, *J* = 8.9 Hz, 1H), 4.32–4.23 (m, 1H), 4.07 (td, *J* = 11.3, 2.2 Hz, 2H), 3.80–3.68 (m, 2H), 3.61 (dd, *J* = 11.4, 8.8 Hz, 1H), 3.49 (s, 3H), 2.36 (s, 3H), 2.08–1.98 (m, 9H) ppm; ^13^C NMR (101 MHz, CDCl3) δ 193.1, 170.6, 170.1, 169.5, 165.8, 165.7, 165.2, 133.4, 133.3, 133.0, 129.9, 129.8, 129.6, 129.3, 129.1, 129.0, 128.5, 128.4, 128.2, 101.2, 96.9, 77.3, 77.0, 76.7, 72.0, 71.7, 71.1, 70.6, 69.6, 69.4, 68.7, 68.3, 62.1, 55.6, 48.5, 30.6, 20.6, 20.6, 20.5 ppm; HRMS (ESI-TOF) *m*/*z* [M + H]+ calcd for [C_42_H_45_O_17_S]+: 853.2377; found: 853.2366.

Methyl 2,3,4-tri-*O*-benzyl-6-*O*-(2-*S*-acetyl-3,4,6-tri-*O*-acetyl-2-thio-*β*-D-glucopyranoside)-*α*-D-glucopyranoside **2b** was synthesized by the use of **2** (50 mg, 0.12 mmol), methyl 2,3,4-tri-*O*-benzyl-*α*-D-glucopyranoside (69 mg, 0.15 mmol), AgOTf (35 mg, 0.14 mmol), and TMSOTf (1.8 uL, 0.01 mmol) through general procedure A. The crude product was purified by flash column chromatography on a silica gel column (EtOAc/petroleum ether = 1:6) to afford **2b** as a colorless oil (86.8 mg, 0.107 mmol, 87% yield). Rf = 0.5 (ethyl acetate/petroleum ether 1:4); ^1^H NMR (600 MHz, CDCl_3_) δ 7.40–7.27 (m, 15H), 5.33 (td, *J* = 9.6, 5.0 Hz, 1H), 5.10–5.03 (m, 1H), 5.01 (d, *J* = 10.7 Hz, 1H), 4.90 (d, *J* = 10.8 Hz, 1H), 4.82 (t, *J* = 11.0 Hz, 2H), 4.76–4.71 (m, 1H), 4.68 (d, *J* = 12.2 Hz, 1H), 4.64 (dd, *J* = 7.3, 3.6 Hz, 2H), 4.28 (dq, *J* = 12.3, 2.2 Hz, 1H), 4.16 (d, *J* = 12.3 Hz, 1H), 4.10 (d, *J* = 10.6 Hz, 1H), 4.03–3.97 (m, 1H), 3.78 (d, *J* = 10.6 Hz, 1H), 3.76–3.71 (m, 2H), 3.60–3.50 (m, 3H), 3.39 (d, *J* = 2.2 Hz, 3H), 2.20 (d, *J* = 2.3 Hz, 3H), 2.10–2.00 (m, 9H) ppm; ^13^C NMR (101 MHz, CDCl_3_) δ 193.0, 170.6, 170.1, 169.4, 138.8, 138.3, 138.1, 128.5, 128.4, 128.3, 128.2, 128.1, 128.0, 127.9, 127.8, 127.7, 127.6, 100.8, 98.2, 98.0, 82.0, 80.0, 79.8, 77.5, 77.4, 77.3, 77.1, 76.7, 75.7, 75.0, 74.9, 73.3, 71.7, 71.1, 69.7, 69.6, 68.2, 62.1, 55.2, 48.7, 30.6, 20.7, 20.6 ppm; HRMS (ESI-TOF) *m*/*z* [M + H]^+^ calcd for [C_42_H_45_O_17_S]^+^: 811.3000; found: 811.3011.

Methyl 2,3,6-tri-*O*-benzyl-4-*O*-(2-*S*-acetyl-3,4,6-tri-*O*-acetyl-2-thio-*β*-D-glucopyranoside)-*α*-D-glucopyranoside **2c** was synthesized by the use of **2** (50 mg, 0.12 mmol), methyl 2,3,6-tri-*O*-benzyl-*α*-D-glucopyranoside (70 mg, 0.15 mmol), AgOTf (35 mg, 0.14 mmol), and TMSOTf (1.8 uL, 0.01 mmol) through general procedure A. The crude product was purified by flash column chromatography on a silica gel column (EtOAc/petroleum ether = 1:6) to afford **2c** as a colorless oil (79.8 mg, 0.098 mmol, 80% yield). Rf = 0.5 (ethyl acetate/petroleum ether 1:4); ^1^H NMR (400 MHz, CDCl_3_) δ 7.46–7.24 (m, 15H), 5.05–4.92 (m, 3H), 4.80 (s, 1H), 4.78–4.71 (m, 2H), 4.66–4.56 (m, 2H), 4.51 (d, *J* = 9.0 Hz, 1H), 4.44 (d, *J* = 12.0 Hz, 1H), 4.15 (dd, *J* = 12.3, 4.3 Hz, 1H), 3.99–3.80 (m, 4H), 3.66 (ddd, *J* = 11.1, 6.8, 2.2 Hz, 2H), 3.53–3.47 (m, 2H), 3.39 (s, 3H), 3.30 (dp, *J* = 6.8, 2.3 Hz, 1H), 2.30 (s, 3H), 2.03–1.94 (m, 9H); ^13^C NMR (101 MHz, CDCl_3_) δ 192.8, 170.6, 170.1, 169.5, 138.7, 138.3, 138.1, 128.5, 128.4, 128.3, 128.2, 128.1, 128.0, 127.8, 127.6, 100.7, 98.2, 98.0, 81.9, 80.1, 79.7, 77.5, 77.4, 77.3, 77.0, 76.0, 75.7, 75.0, 74.9, 73.3, 71.7, 71.1, 69.7, 69.6, 68.2, 62.1, 55.2, 48.7, 30.6, 20.7, 20.6, 20.5 ppm; HRMS (ESI-TOF) *m*/*z* [M + H]^+^ calcd for [C_42_H_51_O_14_S]^+^: 811.2994; found: 811.2971.

Methyl 2-*O*-benzoyl-4,6-*O*-benzylidene-3-*O*-(2-*S*-acetyl-3,4,6-tri-*O*-acetyl-2-thio-*β*-D-glucopyranoside)-*α*-D-glucopyranoside **2e** was synthesized by the use of **2** (50 mg, 0.12 mmol), methyl 2-*O*-benzoyl-4,6-*O*-benzylidene-*α*-D-glucopyranoside (57 mg, 0.15 mmol), AgOTf (35 mg, 0.14 mmol), and TMSOTf (1.8 uL, 0.01 mmol) through general procedure A. The crude product was purified by flash column chromatography on a silica gel column (EtOAc/petroleum ether = 1:4) to afford **2e** as a colorless oil (73.0 mg, 0.099 mmol, 82% yield). Rf = 0.3 (ethyl acetate/petroleum ether 1:4); ^1^H NMR (400 MHz, CDCl_3_) δ 8.15–7.29 (m, 10H), 5.60 (s, 1H), 5.16 (dd, *J* = 9.6, 3.9 Hz, 1H), 5.10–4.93 (m, 3H), 4.77 (d, *J* = 8.9 Hz, 1H), 4.37 (d, *J* = 9.3 Hz, 1H), 4.29 (dd, *J* = 9.9, 4.4 Hz, 1H), 4.19 (dd, *J* = 12.2, 4.2 Hz, 1H), 3.93 (d, *J* = 2.6 Hz, 1H), 3.93–3.86 (m, 1H), 3.81 (t, *J* = 10.1 Hz, 1H), 3.74 (t, *J* = 9.3 Hz, 1H), 3.64–3.53 (m, 2H), 3.37 (s, 3H), 1.95 (d, *J* = 3.8 Hz, 9H), 1.90 (s, 3H); ^13^C NMR (101 MHz, CDCl_3_) δ 192.58, 170.73, 170.11, 169.38, 165.79, 137.33, 133.46, 129.90, 129.76, 129.03, 128.61, 128.11, 126.11, 101.37, 101.34, 97.66, 79.53, 73.12, 71.43, 69.36, 68.89, 62.46, 62.02, 55.40, 48.33, 45.49, 30.13, 29.68, 20.68, 20.55, 20.48 ppm; HRMS (ESI-TOF) *m*/*z* [M + H]^+^ calcd for [C_35_H_41_O_15_S]^+^: 733.2166; found: 733.2138.

Methyl 3,4,6-*O*-benzoyl-2-*O*-(2-*S*-acetyl-3,4,6-tri-*O*-acetyl-2-thio-*β*-D-glucopyranoside)-*β*-D-glucopyranoside **2f** was synthesized by the use of **2** (50 mg, 0.12 mmol), methyl 3,4,6-tri-*O*-benzoyl-*α*-D-glucopyranoside (50 mg, 0.12 mmol), AgOTf (35 mg, 0.14 mmol), and TMSOTf (1.8 uL, 0.01 mmol) through general procedure A. The crude product was purified by flash column chromatography on a silica gel column (EtOAc/petroleum ether = 1:4) to afford **2f** as a colorless oil (89.2 mg, 0.105 mmol, 85% yield). Rf = 0.3 (ethyl acetate/petroleum ether 1:4); ^1^H NMR (400 MHz, CDCl_3_) δ 8.01–7.29 (m, 16H), 5.69 (t, *J* = 9.3 Hz, 1H), 5.53 (t, *J* = 9.7 Hz, 1H), 5.15 (dd, *J* = 11.1, 9.0 Hz, 1H), 5.04 (dd, *J* = 9.9, 8.9 Hz, 1H), 4.87 (d, *J* = 8.9 Hz, 1H), 4.62 (d, *J* = 7.1 Hz, 1H), 4.57 (dd, *J* =12.1, 3.3 Hz, 1H), 4.45 (dd, *J* = 12.1, 5.4 Hz, 1H), 4.29 (dd, *J* = 12.2, 4.3 Hz, 1H), 4.16 (dd, *J* = 12.2, 2.5 Hz, 1H), 4.09–3.99 (m, 1H), 3.89 (dd, *J* = 8.9, 7.1 Hz, 1H), 3.70 (ddd, *J* = 9.9, 4.3, 2.5 Hz, 1H), 3.58 (s, 3H), 3.48 (dd, *J* = 11.2, 8.9 Hz, 1H), 2.09 (s, 3H), 1.99–1.86 (m, 10H); ^13^C NMR (101 MHz, CDCl_3_) δ 193.0, 170.6, 170.0, 169.4, 165.8, 165.7, 165.2, 133.4, 133.3, 133.0, 129.9, 129.7, 129.6, 129.3, 129.1, 129.0, 128.4, 128.3, 128.2, 101.2, 96.9, 77.3, 77.0, 76.7, 72.0, 71.7, 71.1, 70.6, 69.6, 69.4, 68.7, 68.3, 62.0, 55.6, 48.4, 30.6, 20.6, 20.5 ppm; HRMS (ESI-TOF) *m*/*z* [M + H]^+^ calcd for [C_42_H_45_O_17_S]^+^: 853.2377; found: 853.2360.

Phenyl 2-*S*-acetyl-3,4,6-tri-*O*-acetyl-2-thio-*β*-D-glucopyranoside **2g** was synthesized by the use of **2** (50 mg, 0.12 mmol), phenol (15 mg, 0.15 mmol), AgOTf (35 mg, 0.14 mmol), and TMSOTf (1.8 uL, 0.01 mmol) through general procedure A. The crude product was purified by flash column chromatography on a silica gel column (EtOAc/petroleum ether = 1:5) to afford **2g** as a colorless oil (47.7 mg, 0.108 mmol, 88% yield). Rf = 0.4 (ethyl acetate/petroleum ether 1:4); ^1^H NMR (600 MHz, CDCl_3_) δ 7.34–6.98 (m, 5H), 5.47–5.39 (m, 1H), 5.26 (d, *J* = 8.8 Hz, 1H), 5.14 (t, *J* = 9.5 Hz, 1H), 4.34 (dd, *J* = 12.3, 5.4 Hz, 1H), 4.17 (d, *J* = 12.2 Hz, 1H), 3.90 (ddt, *J* = 9.6, 4.7, 2.3 Hz, 1H), 3.85–3.78 (m, 1H), 2.39–2.33 (m, 3H), 2.11–2.03 (m, 9H); ^13^C NMR (101 MHz, CDCl_3_) δ 194.9, 170.3, 169.8, 169.6, 155.8, 129.5, 122.8, 116.6, 99.4, 67.3, 65.9, 65.2, 61.7, 43.7, 30.3, 20.8, 20.7, 20.5 ppm; HRMS (ESI-TOF) *m*/*z* [M + H]^+^ calcd for [C_20_H_25_O_9_S]^+^: 441.1219; found: 441.1215.

Benzyl 2-*S*-acetyl-3,4,6-tri-*O*-acetyl-2-thio-*β*-D-glucopyranoside **2h [19]** was synthesized by the use of **2** (50 mg, 0.12 mmol), benzyl alcohol (16 mg, 0.15mmol), AgOTf (35 mg, 0.14 mmol), and TMSOTf (1.8 uL, 0.01 mmol) through general procedure A. The crude product was purified by flash column chromatography on a silica gel column (EtOAc/petroleum ether = 1:5) to afford **2h** as a colorless oil (48.1 mg, 0.105 mmol, 86% yield). Rf = 0.3 (ethyl acetate/petroleum ether 1:4); ^1^H NMR (600 MHz, CDCl_3_) δ 7.45–7.27 (m, 5H), 5.28 (dd, *J* = 11.3, 9.1 Hz, 1H), 5.09 (t, *J* = 9.5 Hz, 1H), 4.92 (d, *J* = 12.1 Hz, 1H), 4.71 (d, *J* = 8.9 Hz, 1H), 4.64 (d, *J* = 12.2 Hz, 1H), 4.32 (dd, *J* = 12.3, 4.7 Hz, 1H), 4.19 (dd, *J* = 12.3, 2.3 Hz, 1H), 3.72 (ddd, *J* = 10.2, 4.7, 2.3 Hz, 1H), 3.64 (dd, *J* = 11.3, 9.0 Hz, 1H), 2.31 (d, *J* = 1.6 Hz, 3H), 2.13 (d, *J* = 1.8 Hz, 3H), 2.03 (d, *J* = 1.7 Hz, 6H). The data are identical to those in the literature [19].

*p*-Methoxyphenol 2-*S*-acetyl-3,4,6-tri-*O*-acetyl-2-thio-*β*-D-glucopyranoside **2i** was synthesized by the use of **2** (50 mg, 0.12 mmol), *p*-methoxyphenol (18 mg, 0.15mmol), AgOTf (35 mg, 0.14 mmol), and TMSOTf (1.8 uL, 0.01 mmol) through general procedure A. The crude product was purified by flash column chromatography on a silica gel column (EtOAc/petroleum ether = 1:5) to afford **2h** as a colorless oil (50.4 mg, 0.107 mmol, 87% yield). Rf = 0.4 (ethyl acetate/petroleum ether 1:4); ^1^H NMR (400 MHz, CDCl_3_) δ 7.32–6.78 (m, 4H), 5.38 (dd, *J* = 11.3, 9.0 Hz, 1H), 5.13 (d, *J* = 9.8 Hz, 1H), 5.10 (s, 1H), 4.16 (dd, *J* = 12.2, 2.5 Hz, 1H), 3.83 (ddd, *J* = 10.0, 5.2, 2.4 Hz, 1H), 3.78 (s, 3H), 2.36 (s, 2H), 2.09 (s, 3H), 2.04 (d, *J* = 3.8 Hz, 6H); ^13^C NMR (101 MHz, CDCl_3_) δ 192.9, 170.5, 170.0, 169.4, 155.7, 151.0, 118.8, 114.5, 100.5, 92.1, 71.8, 71.1, 69.5, 68.9, 62.1, 61.6, 55.6, 48.7, 47.1, 30.7, 30.6, 20.6, 20.5 ppm; HRMS (ESI-TOF) *m*/*z* [M + H]^+^ calcd for [C_21_H_27_O_10_S]^+^: 471.1319; found: 471.1309.

Methyl 2,3,4-tri-*O*-benzoyl-6-*O*-(2-*S*-acetyl-3,4,6-tri-*O*-benzoyl-2-thio-*β*-D-galactopyranoside)-*α*-D-glucopyranoside **3a** was synthesized by the use of **3** (80 mg, 0.13 mmol), methyl 2,3,4-tri-*O*-benzoyl-*α*-D-glucopyranoside (79 mg, 0.16 mmol), AgOTf (35 mg, 0.14 mmol), and TMSOTf (1.8 uL, 0.01 mmol) through general procedure A. The crude product was purified by flash column chromatography on a silica gel column (EtOAc/petroleum ether = 1:4) to afford **3a** as a colorless oil (126.2 mg, 0.122 mmol, 90% yield). Rf = 0.3 (ethyl acetate/petroleum ether 1:4); ^1^H NMR (600 MHz, CDCl_3_) δ 8.15–7.27 (m, 30H), 6.39 (d, *J* = 2.0 Hz, 1H), 6.11 (t, *J* = 9.9 Hz, 1H), 5.87–5.76 (m, 1H), 5.60 (t, *J* = 9.9 Hz, 1H), 5.28 (s, 1H), 5.21–5.08 (m, 2H), 4.54–4.46 (m, 2H), 4.38–4.32 (m, 1H), 4.21 (ddd, *J* = 10.3, 4.9, 2.1 Hz, 1H), 3.98 (dd, *J* = 11.6, 4.9 Hz, 1H), 3.78 (dd, *J* = 11.5, 2.2 Hz, 1H), 3.47 (s, 3H), 2.43 (s, 3H); ^13^C NMR (101 MHz, CDCl_3_) δ 166.4, 165.8, 165.2, 133.7, 133.6, 133.5, 133.4, 133.2, 133.2, 130.1, 128.6, 129.9, 129.8, 129.7, 129.6, 129.4, 129.2, 129.0, 128.9, 128.5, 128.4, 128.3, 128.2, 101.6, 97.1, 96.9, 77.2, 75.4, 72.0, 71.2, 70.6, 70.4, 70.1, 69.7, 69.5, 69.4, 68.8, 67.3, 62.0, 61.0, 60.4, 55.6, 45.7, 39.5, 30.7, 21.0, 14.2; HRMS (ESI-TOF) *m*/*z* [M + H]^+^ calcd for [C_57_H_51_O_17_S]^+^: 1039.2841; found: 1039.2834.

Methyl 2,3,4-tri-*O*-benzyl-6-*O*-(2-*S*-acetyl-3,4,6-tri-*O*-benzoyl-2-thio-*β*-D-galactopyranoside)-*α*-D-glucopyranoside **3b** was synthesized by the use of **3** (80 mg, 0.13 mmol) with methyl 2,3,4-tri-*O*-benzyl-*α*-D-glucopyranoside (73 mg, 0.16 mmol), AgOTf (35 mg, 0.14 mmol), and TMSOTf (1.8 uL, 0.01 mmol) through general procedure A. The crude product was purified by flash column chromatography on a silica gel column (EtOAc/petroleum ether = 1:4) to afford **3b** as a colorless oil (118.4 mg, 0.119 mmol, 88% yield). Rf = 0.3 (ethyl acetate/petroleum ether 1:4); ^1^H NMR (400 MHz, CDCl_3_) δ 8.16–7.07 (m, 30H), 6.44 (dd, *J* = 9.9, 7.8 Hz, 1H), 6.15 (td, *J* = 9.8, 4.6 Hz, 1H), 5.39 (ddd, *J* = 10.3, 9.4, 3.5 Hz, 1H), 5.29–5.13 (m, 2H), 4.94 (dd, *J* = 11.6, 8.7 Hz, 1H), 4.78–4.47 (m, 4H), 4.47–4.34 (m, 3H), 4.11–3.94 (m, 3H), 3.77–3.52 (m, 4H), 3.43 (s, 3H), 2.58 (s, 3H); ^13^C NMR (101 MHz, CDCl_3_) δ 170.7, 166.0, 165.7, 165.0, 138.6, 138.0, 137.9, 137.9, 137.90, 133.7, 133.7, 133.6, 133.3, 130.3, 130.1, 130.0, 129.7, 129.3, 128.7, 128.5, 128.4, 128.1, 128.0, 127.9, 127.8, 127.7, 100.6, 98.6, 98.0, 82.0, 77.2, 75.8, 75.0, 74.4, 73.4, 72.1, 72.0, 71.8, 71.2, 68.6, 67.8, 65.4, 65.0, 63.1, 61.8, 60.4, 57.4, 55.2, 29.7, 20.8, 14.2 ppm; HRMS (ESI-TOF) *m*/*z* [M + H]^+^ calcd for [C_57_H_57_O_14_S]^+^: 997.3464; found: 997.3450.

Methyl 2,3,6-tri-*O*-benzoyl-4-*O*-(2-*S*-acetyl-3,4,6-tri-*O*-benzoyl-2-thio-*β*-D-galactopyranoside)-*α*-D-glucopyranoside **3d** was synthesized by the use of **3** (80 mg, 0.13 mmol), methyl 2,3,6-tri-*O*-benzoyl-*α*-D-glucopyranoside (79 mg, 0.16 mmol), AgOTf (35 mg, 0.14 mmol), and TMSOTf (1.8 uL, 0.01 mmol) through general procedure A. The crude product was purified by flash column chromatography on a silica gel column (EtOAc/petroleum ether = 1:4) to afford **3d** as a colorless oil (116.4 mg, 0.112 mmol, 83% yield). Rf = 0.3 (ethyl acetate/petroleum ether 1:4); ^1^H NMR (400 MHz, CDCl_3_) δ 8.23–7.23 (m, 30H), 6.23–6.11 (m, 1H), 5.87 (d, *J* = 3.4 Hz, 1H), 5.61–5.51 (m, 2H), 5.30–5.25 (m, 2H), 4.91 (d, *J* = 9.0 Hz, 1H), 4.58 (dd, *J* = 11.3, 6.4 Hz, 1H), 4.46–4.12 (m, 5H), 3.86 (dd, *J* = 11.4, 6.5 Hz, 1H), 3.54 (s, 3H), 2.27 (s, 3H); ^13^C NMR (151 MHz, CDCl_3_) δ 215.2, 138.1, 137.9, 137.7, 133.6, 132.1, 128.9, 128.4, 128.3, 127.9, 127.8, 127.7, 127.6, 86.4, 84.6, 79.9, 79.4, 77.6, 75.4, 75.2, 73.5, 68.9, 29.7, 19.5 ppm; HRMS (ESI-TOF) *m*/*z* [M + H]^+^ calcd for [C_57_H_51_O_17_S]^+^: 1039.2841; found: 1039.2830.

Phenyl 2-*S*-acetyl-3,4,6-tri-*O*-acetyl-2-thio-*β*-D-galactopyranoside **3g** was synthesized by the use of **3** (80 mg, 0.13 mmol) with phenol (15 mg, 0.16 mmol), AgOTf (35 mg, 0.14 mmol), and TMSOTf (1.8 uL, 0.01 mmol) through general procedure A. The crude product was purified by flash column chromatography on a silica gel column (EtOAc/petroleum ether = 1:4) to afford **3g** as a colorless oil (71.1 mg, 0.113 mmol, 84% yield). Rf = 0.3 (ethyl acetate/petroleum ether 1:5); ^1^H NMR (600 MHz, CDCl_3_) δ 8.15–7.32 (m, 20H), 5.95 (dd, *J* = 9.3, 4.7 Hz, 1H), 5.43 (dd, *J* = 3.7, 1.7 Hz, 1H), 5.05 (d, *J* = 1.6 Hz, 1H), 4.87 (d, *J* = 12.1 Hz, 1H), 4.70 (d, *J* = 4.4 Hz, 1H), 4.55 (dd, *J* = 11.5, 7.9 Hz, 1H), 4.49–4.44 (m, 1H), 2.26 (s, 3H) ppm; ^13^C NMR (101 MHz, CDCl_3_) δ 194.9, 164.9, 165.7, 156.2, 133.3, 133.1, 131.0, 129.6, 129.5, 129.4, 129.2, 129.1, 128.7, 128.4, 128.3, 123.2, 116.6, 98.1, 68.8, 68.3, 62.9, 43.4, 30.4 ppm; HRMS (ESI-TOF) *m*/*z* [M + H]^+^ calcd for [C_57_H_51_O_17_S]^+^: 627.1683; found: 627.1608.

Methyl 2,3,4-tri-*O*-benzoyl-6-*O*-(3,4,6-tri-*O*-acetyl-2-deoxy-*β*-D-glucopyranoside)-*α*-D-glucopyranoside **2aD** was synthesized from **2a** (90 mg, 0.106 mmol) through general procedure B, where DMF (0.8 mL), hydrated hydrazine (9.0 μL, 0.19 mmol), TCEP·HCl (40 mg, 0.15 mmol), and Ru(bpy)_3_Cl_2_ (2 mg, 0.002 mmol) were used. The crude product was purified by chromatography on a silica gel column (EtOAc/petroleum ether = 1:4) to afford **2aD** as a colorless oil (72.3 mg, 0.093 mmol, 88% yield). Rf = 0.3 (ethyl acetate/petroleum ether 1:4); ^1^H NMR (400 MHz, CDCl_3_) δ 8.04–7.29 (m, 16H), 6.17 (t, *J* = 9.7 Hz, 1H), 5.57 (t, *J* = 9.9 Hz, 1H), 5.57 (t, *J* = 9.9 Hz, 1H), 5.26 (d, *J* = 3.7 Hz, 2H), 5.04 (dd, *J* = 11.4, 5.3 Hz, 1H), 4.97 (t, *J* = 9.5 Hz, 1H), 4.59 (dd, *J* = 9.8, 2.0 Hz, 1H), 4.33–4.21 (m, 2H), 4.09 (ddd, *J* = 20.8, 11.7, 2.2 Hz, 2H), 3.68 (dd, *J* = 11.3, 5.9 Hz, 1H), 3.58 (ddd, *J* = 9.6, 5.0, 2.4 Hz, 1H), 3.49 (s, 3H), 2.05 (s, 3H), 2.04 (s, 6H); ^13^C NMR (151 MHz, CDCl_3_) δ 170.78, 170.28, 169.80, 165.77, 165.38, 133.52, 133.36, 133.10, 129.94, 129.86, 129.66, 129.22, 129.07, 128.88, 128.50, 128.42, 128.28, 100.40, 96.94, 72.04, 71.95, 70.44, 69.31, 69.14, 68.72, 68.34, 62.42, 55.55, 31.94, 29.71, 29.67, 29.37, 22.70, 20.91, 20.72, 14.13; HRMS (ESI-TOF) *m*/*z* [M + H]^+^ calcd for [C_57_H_51_O_17_S]^+^: 1039.2841; found: 1039.2830.

Methyl 2,3,4-tri-*O*-benzyl-6-*O*-(3,4,6-tri-*O*-acetyl-2-deoxy-*β*-D-glucopyranoside)-*α*-D-glucopyranoside **2bD** [41] was synthesized from **2b** (90 mg, 0.111 mmol) through general procedure B, where DMF (0.8 mL), hydrated hydrazine (9.0 μL, 0.19 mmol), TCEP·HCl (40 mg, 0.15 mmol), and Ru(bpy)_3_Cl_2_ (2 mg, 0.002 mmol) were used. The crude product was purified by chromatography on a silica gel column (EtOAc/petroleum ether = 1:4) to afford **2bD** as a colorless oil (71.1 mg, 0.097 mmol, 87% yield). Rf = 0.5 (ethyl acetate/petroleum ether 1:4); ^1^H NMR (400 MHz, CDCl_3_) δ 8.09–7.11 (m, 15H), 5.01 (d, *J* = 2.5 Hz, 1H), 5.00 (s, 1H), 4.98 (s, 2H), 4.95 (d, *J* = 3.4 Hz, 2H), 4.85 (s, 1H), 4.84–4.83 (m, 2H), 4.82 (d, *J* = 4.3 Hz, 2H), 4.76 (s, 1H), 4.70 (t, *J* = 3.8 Hz, 2H), 4.36 (d, *J* = 8.5 Hz, 1H), 4.32 (d, *J* = 8.6 Hz, 2H), 4.14 (dd, *J* = 3.9, 2.2 Hz, 1H), 4.10 (dd, *J* = 5.4, 3.4 Hz, 2H), 4.01 (d, *J* = 8.1 Hz, 1H), 3.75 (d, *J* = 6.7 Hz, 1H), 3.67–3.62 (m, 2H), 3.61 (d, *J* = 6.0 Hz, 1H), 3.58–3.53 (m, 1H), 3.41 (s, 3H), 2.18 (d, *J* = 4.6 Hz, 1H), 2.09 (d, *J* = 6.7 Hz, 3H), 2.06 (d, *J* = 3.6 Hz, 3H), 2.03 (s, 3H). The data are identical to those in the literature [42].

Methyl 2,3,6-tri-*O*-benzyl-2-*O*-(3,4,6-tri-*O*-acetyl-2-deoxy-*β*-D-glucopyranoside)-*α*-D-glucopyranoside **2cD** [41] was synthesized from **2c** (90 mg, 0.111 mmol) through general procedure C, where DMF (0.8 mL), hydrated hydrazine (9.0 μL, 0.19 mmol), TCEP·HCl (40 mg, 0.15 mmol), and Ru(bpy)_3_Cl_2_ (2 mg, 0.002 mmol) were used. The crude product was purified by chromatography on a silica gel column (EtOAc/petroleum ether = 1:4) to afford **2cD** as a colorless oil (65.4 mg, 0.089 mmol, 80% yield). Rf = 0.5 (ethyl acetate/petroleum ether 1:4); ^1^H NMR (400 MHz, CDCl_3_) δ 7.50–7.29 (m, 15H), 4.97 (d, *J* = 11.4 Hz, 1H), 4.90 (t, *J* = 9.5 Hz, 1H), 4.80–4.76 (m, 1H), 4.77–4.72 (m, 2H), 4.63–4.57 (m, 2H), 4.41 (d, *J* = 12.0 Hz, 1H), 4.25 (d, *J* = 8.6 Hz, 1H), 4.16 (td, *J* = 12.5, 3.6 Hz, 2H), 4.02 (dd, *J* = 10.0, 8.9 Hz, 1H), 3.93 (dd, *J* = 12.3, 2.4 Hz, 1H), 3.86 (t, *J* = 9.2 Hz, 1H), 3.75 (dt, *J* = 10.1, 2.3 Hz, 1H), 3.66 (dd, *J* = 10.8, 1.9 Hz, 1H), 3.52 (dd, *J* = 9.6, 3.7 Hz, 1H), 3.40 (s, 3H), 3.30 (ddd, *J* = 10.0, 4.4, 2.4 Hz, 1H), 2.11–1.94 (m, 9H).

Methyl 2-*O*-benzoyl-4,6-*O*-benzylidene-3-*O*-(3,4,6-tri-*O*-acetyl-2-deoxy-*β*-D-glucopyranoside)-*α*-D-glucopyranoside **2eD** was synthesized from **2e** (90 mg, 0.123 mmol) through general procedure C, where DMF (0.8 mL), hydrated hydrazine (9.0 μL, 0.19 mmol), TCEP·HCl (40 mg, 0.15 mmol), and Ru(bpy)_3_Cl_2_ (2 mg, 0.002 mmol) were used. The crude product was purified by chromatography on a silica gel column (EtOAc/petroleum ether = 1:4) to afford **2eD** as a colorless oil (67.1 mg, 0.102 mmol, 83% yield). Rf = 0.3 (ethyl acetate/petroleum ether 1:4); ^1^H NMR (400 MHz, CDCl_3_) δ 8.12–7.30 (m, 12H), 5.58 (s, 1H), 5.12 (dd, *J* = 9.4, 3.9 Hz, 1H), 5.08 (d, *J* = 3.9 Hz, 1H), 4.90 (dd, *J* = 10.0, 8.9 Hz, 1H), 4.68 (dt, *J* = 8.9, 5.7 Hz, 2H), 4.48 (t, *J* = 9.3 Hz, 1H), 4.32 (dd, *J* = 10.1, 4.6 Hz, 1H), 4.16 (dd, *J* = 12.3, 4.1 Hz, 1H), 4.00–3.90 (m, 2H), 3.83 (t, *J* = 10.3 Hz, 1H), 3.70 (t, *J* = 9.3 Hz, 1H), 3.49–3.43 (m, 1H), 3.41 (s, 3H), 2.78 (dd, *J* = 11.5, 8.9 Hz, 1H), 1.95 (d, *J* = 7.6 Hz, 6H), 1.83 (s, 3H); ^13^C NMR (101 MHz, CDCl_3_) δ 170.66, 170.00, 169.50, 165.77, 165.26, 137.79, 133.53, 133.37, 133.14, 133.10, 129.93, 129.90, 129.85, 129.68, 129.18, 129.04, 128.99, 128.80, 128.49, 128.42, 128.27, 99.53, 96.84, 72.11, 70.44, 70.29, 69.42, 68.77, 68.23, 66.33, 66.15, 62.22, 56.47, 55.57, 21.04, 20.72, 20.71, 20.69.; HRMS (ESI-TOF) *m*/*z* [M + H]^+^ calcd for [C_33_H_39_O_14_]^+^: 659.2334; found: 659.2329.

Methyl 3,4,6-tri-*O*-benzoyl-2-*O*-(3,4,6-tri-*O*-acetyl-*β*-D-glucopyranoside)-*β*-D-glucopyranoside **2fD** was synthesized from **2f** (90 mg, 0.105 mmol) through general procedure B, where DMF (0.8 mL), hydrated hydrazine (9.0 μL, 0.19 mmol), TCEP·HCl (40 mg, 0.15 mmol), and Ru(bpy)_3_Cl_2_ (2 mg, 0.002 mmol) were used. The crude product was purified by chromatography on a silica gel column (EtOAc/petroleum ether = 1:4) to afford **2fD** as a colorless oil (69.9 mg, 0.089 mmol, 85% yield). Rf = 0.3 (ethyl acetate/petroleum ether 1:4); ^1^H NMR (400 MHz, CDCl_3_) δ 8.05–7.30 (m, 15H), 5.78 (t, *J* = 9.2 Hz, 1H), 5.60 (td, *J* = 9.6, 2.0 Hz, 1H), 5.02–4.95 (m, 1H), 4.89 (dd, *J* = 11.0, 9.2 Hz, 1H), 4.69–4.64 (m, 1H), 4.63 (d, *J* = 2.7 Hz, 1H), 4.60 (d, *J* = 3.3 Hz, 1H), 4.51–4.45 (m, 1H), 4.28 (dd, *J* = 12.3, 4.3 Hz, 1H), 4.16–4.06 (m, 1H), 4.04 (dd, *J* = 8.8, 7.1 Hz, 1H), 3.92 (dd, *J* = 8.5, 7.0 Hz, 1H), 3.73–3.65 (m, 1H), 3.63 (s, 1H), 3.60 (d, *J* = 2.8 Hz, 3H), 2.08 (d, *J* = 4.7 Hz, 3H), 2.00 (d, *J* = 4.2 Hz, 6H); ^13^C NMR (101 MHz, CDCl_3_) δ 170.1, 169.9, 169.5, 166.1, 165.6, 165.3, 133.3, 133.2, 133.0, 129.8, 129.7, 129.6, 129.3, 128.8, 128.5, 128.4, 128.3, 103.9, 102.9, 102.6, 99.8, 79.7, 78.8, 74.2, 72.1, 71.9, 71.7, 70.2, 69.7, 69.5, 69.1, 68.8, 62.0, 57.0, 20.7, 20.5; HRMS (ESI-TOF) *m*/*z* [M + H]^+^ calcd for [C_40_H_43_O_16_]^+^: 779.2546; found: 779.2531.

Phenyl 3,4,6-tri-*O*-acetyl-2-deoxy-*β*-D-glucopyranoside **2gD [19]** was synthesized from **2g** (50 mg, 0.113 mmol) through general procedure B, where DMF (0.8 mL), hydrated hydrazine (9.0 μL, 0.19 mmol), TCEP·HCl (40 mg, 0.15 mmol), and Ru(bpy)_3_Cl_2_ (2 mg, 0.002 mmol) were used. The crude product was purified by chromatography on a silica gel column (EtOAc/petroleum ether = 1:4) to afford **2gD** as a colorless oil (34.9 mg, 0.095 mmol, 84% yield). Rf = 0.4 (ethyl acetate/petroleum ether 1:4); ^1^H NMR (400 MHz, CDCl_3_) δ 7.41–6.97 (m, 5H), 5.22 (dd, *J* = 9.6, 2.2 Hz, 1H), 5.19–5.02 (m, 2H), 4.34 (dd, *J* = 12.2, 5.5 Hz, 1H), 4.16 (dt, *J* = 12.2, 2.3 Hz, 1H), 3.80 (ddd, *J* = 8.8, 5.7, 2.4 Hz, 1H), 2.54 (ddd, *J* = 12.8, 5.0, 2.2 Hz, 1H), 2.16–2.03 (m, 10H). The data are identical to those in the literature [19].

Benzyl 3,4,6-tri-*O*-acetyl-2-deoxy-*β*-D-glucopyranoside **2hD [42]** was synthesized from **2h** (50 mg, 0.110 mmol) through general procedure B, where DMF (0.8 mL), hydrated hydrazine (9.0 μL, 0.19 mmol), TCEP·HCl (40 mg, 0.15 mmol), and Ru(bpy)_3_Cl_2_ (2 mg, 0.002 mmol) were used. The crude product was purified by chromatography on a silica gel column (EtOAc/petroleum ether = 1:4) to afford **2hD** as a colorless oil (34.7 mg, 0.091 mmol, 83% yield). Rf = 0.3 (ethyl acetate/petroleum ether 1:4); ^1^H NMR (400 MHz, CDCl_3_) δ 7.51–7.33 (m, 6H), 5.15 (dd, *J* = 11.2, 8.9 Hz, 1H), 4.99–4.93 (m, 2H), 4.56 (d, *J* = 8.6 Hz, 1H), 4.24 (dd, *J* = 12.2, 4.9 Hz, 1H), 4.10 (dd, *J* = 12.2, 2.4 Hz, 1H), 3.46 (ddd, *J* = 10.2, 5.0, 2.5 Hz, 1H), 2.93 (dd, *J* = 11.2, 8.6 Hz, 1H), 2.32–2.18 (m, 1H), 2.10–2.03 (m, 9H). The data are identical to those in the literature [43].

*p*-Methoxylphenyl 3,4,6-tri-*O*-acetyl-2-deoxy-*β*-D-glucopyranoside **2iD** [19] was synthesized from **2i** (50 mg, 0.106 mmol) through general procedure B, where DMF (0.8 mL), hydrated hydrazine (9.0 μL, 0.19 mmol), TCEP·HCl (40 mg, 0.15 mmol), and Ru(bpy)_3_Cl_2_ (2 mg, 0.002 mmol) were used. The crude product was purified by chromatography on a silica gel column (EtOAc/petroleum ether = 1:4) to afford **2iD** as a colorless oil (35.6 mg, 0.091 mmol, 85% yield). Rf = 0.4 (ethyl acetate/petroleum ether 1:4); ^1^H NMR (600 MHz, CHCl_3_) δ 7.51–7.37 (m, 2H), 6.79–6.65 (m, 2H), 5.56 (dd, *J* = 3.7, 1.3 Hz, 1H), 5.50 (ddd, *J* = 11.6, 9.4, 5.4 Hz, 1H), 5.15 (t, *J* = 9.8 Hz, 1H), 4.24 (dd, *J* = 12.3, 4.8 Hz, 1H), 4.10 (dd, *J* = 12.2, 2.4 Hz, 1H), 3.87 (s, 3H), 3.46 (ddd, *J* = 10.2, 5.0, 2.5 Hz, 1H), 2.54 (ddd, *J* = 13.1, 5.4, 1.4 Hz, 1H), 2.15 (s, 6H), 2.04 (s, 3H) ppm. The data are identical to those in the literature [19].

Methyl 3,4,6-tri-*O*-acetyl-*β*-D-mannopyranoside **5** [43]. Methyl 3,4,6-tri-*O*-acetyl-*β*-D-glucopyranoside **8** (320 mg, 1.0 mmol) in DCM (4 mL) was added to pyridine (0.7 mL) at −20 °C. Tf_2_O (0.34 mL, 1.99 mmol) was added dropwise, and the mixture was stirred while allowing it to warm from −20 °C to 10 °C for 2 h. The resulting mixture was diluted with dichloromethane and washed with 1 M HCl, saturated aqueous NaHCO_3_, water, and brine. The collected organic phase was dried with MgSO_4_ and then concentrated under reduced pressure. The crude product was dissolved in toluene (2 mL), and TBANO_2_ (1440 mg, 5 mmol) was added and then allowed to react at room temperature for 6 h. Then, the mixture was concentrated and purified by flash column chromatography (EtOAc/hexane = 1:1), yielding **5** as a white solid (256 mg, 0.80 mmol, 80% yield). Rf = 0.3 (ethyl acetate/petroleum ether 2:1); ^1^H NMR (400 MHz, CDCl_3_) δ 5.40 (t, *J* = 9.8 Hz, 1H), 5.00 (dd, *J* = 9.8, 3.1 Hz, 1H), 4.52 (d, *J* = 1.0 Hz, 1H), 4.33 (dd, *J* = 12.2, 5.0 Hz, 1H), 4.21–4.13 (m, 2H), 3.65 (ddd, *J* = 9.8, 5.0, 2.6 Hz, 1H), 3.59 (s, 3H), 2.39 (s, 1H), 2.13 (s, 3H), 2.11 (s, 3H), 2.06 (s, 3H). The data are identical to those in the literature [41].

Methyl 3,4,6-tri-O-benzoyl-*β*-D-galactopyranoside **6 [43]** was obtained starting from 3,4,6-tri-*O*-benzoyl-galactal (160 mg, 0.35 mmol) through general procedure C to obtain the crude 1,2-anhydro sugar. The crude 1,2-anhydro sugar was then dissolved in anhydrous CH_3_OH (2 mL) and kept at rt until TLC showed a complete consumption of the starting material (ca. 2 h). The reaction mixture was concentrated and purified by flash column chromatography (EtOAc/hexane = 1:3) to obtain **6** as a white solid (148.5 mg, 84% yield). Rf = 0.3 (ethyl acetate/petroleum ether 1:2); ^1^H NMR (400 MHz, CDCl_3_) δ 8.14–7.30 (m, 16H), 5.94 (dd, *J* = 3.6, 1.2 Hz, 1H), 5.41 (dd, *J* = 10.2, 3.5 Hz, 1H), 4.69 (dd, *J* = 11.2, 6.6 Hz, 1H), 4.50 (d, *J* = 7.7 Hz, 1H), 4.39 (dd, *J* = 11.3, 6.8 Hz, 1H), 4.27 (td, *J* = 6.6, 1.2 Hz, 1H), 4.13 (dd, *J* = 10.1, 7.7 Hz, 1H), 3.69 (s, 3H). The data are identical to those in the literature [41].

Methyl 3,4,6-tri-*O*-acetyl-*β*-D-glucopyranoside **8 [39]** was obtained starting from 3,4,6-tri-*O*-acetyl-glucal (100 mg, 0.37 mmol) through general procedure C to obtain the crude 1,2-anhydro sugar. The crude 1,2-anhydro sugar was then dissolved in anhydrous CH_3_OH (2 mL) and kept at rt until TLC showed a complete consumption of the starting material (ca. 2 h). The reaction mixture was concentrated and purified by flash column chromatography (EtOAc/hexane = 1:1) to obtain **8** as a white solid (107.1 mg,0.334 mmol, 91%). Rf = 0.3 (ethyl acetate/petroleum ether 1:1); ^1^H NMR (600 MHz, CDCl_3_) δ 5.14–5.07 (m, 1H), 5.01 (td, *J* = 9.8, 2.4 Hz, 1H), 4.31–4.24 (m, 2H), 4.11 (d, *J* = 11.9 Hz, 1H), 3.69 (ddd, *J* = 10.2, 4.6, 2.2 Hz, 1H), 3.59–3.53 (m, 3H), 2.92 (s, 1H), 2.09–2.00 (m, 9H). The data are identical to those in the literature [39].

## 4. Conclusions

The stereocontrolled synthesis of 2-deoxy β-glycosides is more challenging compared to that of 2-deoxy α-glycosides in carbohydrate chemistry. The indirect approach is the most commonly used approach to obtain 2-deoxy glycosides with an exclusive β-configuration. The thioacetyl group has particular advantages compared to other directing groups, such as an excellent neighboring group participation effect in stereocontrolled glycosylation and easy removal by light-induced desulfurization. In this study, we developed an indirect method for the synthesis of 2-deoxyglycosides with an exclusive β-configuration by the use of 2-SAc glycosyl bromide donors. The glycosylation of such donors with various acceptors under an improved Koenigs–Knorr condition afforded glycosylation products with an exclusive β-configuration in excellent yields, and then 2-deoxyglycoside derivatives with an exclusive β-configuration were efficiently obtained by desulfurization under blue light. We also investigated the synthetic approach of 2-SAc glycosyl donors using glycals as the starting materials. Methyl 2-OH glycosides can be efficiently synthesized by the oxidation of these glycals and subsequent reaction of the 1,2-anhydro products with methanol, and then they can be further converted to various 2-SAc glycosyl donors by introducing thioacetate into two positions (Scheme 2c). With efficient access to 2-SAc glycosyl donors, the synthetic method of 2-deoxyglycosides through desulfurization will have more practical applications.

## Figures and Tables

**Table 1:** The optimization of the glycosylation conditions using the 2-SAc glucosyl bromide donor ^a^.

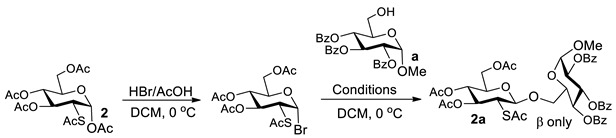		
Entry	Various Conditions (equiv)	Yields %
1	Ag_2_O (0.5), TfOH (0.4), 30 min	65%
2	Ag_2_O (1.0), TfOH (0.4), 30 min	78%
3	AgOTf (2.0), TfOH (0.4), 10 min	88%
4	AgOTf (1.0), TfOH (0.4), 60 min	75%
5	AgOTf (1.2), TfOH (0.4), 10 min	85%
6	AgOTf (1.2), TfOH (0.1), 10 min	87%
7	AgOTf (1.2), TMSOTf (0.1), 10 min	89%
8	AgOTf (1.2), 6 h	64%

^a^ Reaction conditions: the crude 2-SAc glucosyl bromide donor (0.1 M in dry CH_2_Cl_2_), **a** (1.2 equiv), and a 4 Å molecular sieve.

## Data Availability

The data are contained within the article and Supplementary Materials.

## Supplementary Materials

The following supporting information can be downloaded at https://www.mdpi.com/article/10.3390/molecules30010185/s1, Figures S1–S28: ^1^H and ^13^C NMR spectra of compounds **2a**, **2b**, **2c**, **2e**, **2f**, **2g**, **2i**, **3a**, **3b**, **3d**, **3g**, **2aD**, **2eD**, **2fD**.

## References

[1] Ohta T., Hashimoto E., Hasegawa M.J. (1992). Cloning and analysis of a gene (SMS13) encoding sannamycin B-glycyltransferase from Streptomyces sannanensis and its distribution among actinomycetes. J. Antibiot..

[2] Langenhan J.M., Griffith B.R., Thorson J.S. (2005). Neoglycorandomization and Chemoenzymatic Glycorandomization:  Two Complementary Tools for Natural Product Diversification. J. Nat. Prod..

[3] Xu C.-D., Zhang H.-J., Ma Z.-J. (2019). Pyrimidine Nucleosides from *Streptomyces* sp. SSA28. J. Nat. Prod..

[4] Balmond E.I., Benito-Alifonso D., Coe D.M., Alder R.W., McGarrigle E.M., Galan M.C. (2014). A 3,4-*trans*-Fused Cyclic Protecting Group Facilitates *α*-Selective Catalytic Synthesis of 2-Deoxyglycosides. Angew. Chem. Int. Ed..

[5] Sau A., Williams R., Palo-Nieto C., Franconetti A., Medina S., Galan M.C. (2017). Palladium-Catalyzed Direct Stereoselective Synthesis of Deoxyglycosides from Glycals. Angew. Chem. Int. Ed..

[6] Mizia J.C., Bennett C.S. (2019). Reagent Controlled Direct Dehydrative Glycosylation with 2-Deoxy Sugars: Construction of the Saquayamycin Z Pentasaccharide. Org. Lett..

[7] Romeo J.R., McDermott L., Bennett C.S. (2020). Reagent-Controlled *α*-Selective Dehydrative Glycosylation of 2,6-Dideoxy Sugars: Construction of the Arugomycin Tetrasaccharide. Org. Lett..

[8] Liu M., Liu K.-M., Xiong D.-C., Zhang H., Li T., Li B., Qin X., Bai J., Ye X.-S. (2020). Stereoselective Electro-2-deoxyglycosylation from Glycals. Angew. Chem. Int. Ed..

[9] Issa J.P., Bennett C.S. (2014). A Reagent-Controlled S_N_2-Glycosylation for the Direct Synthesis of *β*-Linked 2-Deoxy-Sugars. J. Am. Chem. Soc..

[10] Zhu D., Baryal K.N., Adhikari S., Zhu J. (2014). Direct Synthesis of 2-Deoxy-*β*-Glycosides via Anomeric *O*-Alkylation with Secondary Electrophiles. J. Am. Chem. Soc..

[11] Zhang Q., Dong H. (2024). Recent Advance in the β-Stereoselective Synthesis of 2-Deoxy Glycosides. Asian J. Org. Chem..

[12] Bennett C.S., Galan M.C. (2018). Methods for 2-Deoxyglycoside Synthesis. Chem. Rev..

[13] Kövér A., Boutureira O., Matheu M.I., Díaz Y., Castillón S. (2014). Tuning the Stereoelectronic Properties of 1-Sulfanylhex-1-enitols for the Sequential Stereoselective Synthesis of 2-Deoxy-2-iodo-*β*-D-allopyranosides. J. Org. Chem..

[14] Mukherjee M.M., Ghosh R., Hanover J.A. (2022). Recent Advances in Stereoselective Chemical O-Glycosylation Reactions. Front. Mol. Biosci..

[15] Yang D.-M., Chen Y., Sweeney R.P., Lowary T.L., Liang X.-Y. (2018). Stereocontrolled Synthesis of 2-Deoxy-galactopyranosides via Isopropylidene-Protected 6-O-Silylated Donors. Org. Lett..

[16] Meng S., Zhong W., Yao W., Li Z. (2020). Stereoselective Phenylselenoglycosylation of Glycals Bearing a Fused Carbonate Moiety toward the Synthesis of 2-Deoxy-β-galactosides and β-Mannosides. Org. Lett..

[17] Knapp S., Kirk B.A. (2003). Glycosylation with 2-thio-*S*-acetyl participation. Tetrahedron Lett..

[18] Ge J.-T., Li Y.-Y., Tian J., Liao R.-Z., Dong H. (2017). Synthesis of Deoxyglycosides by Desulfurization under UV Light. J. Org. Chem..

[19] Ge J.-T., Zhou L., Luo T., Lv J., Dong H. (2019). A One-Pot Method for Removal of Thioacetyl Group via Desulfurization under Ultraviolet Light to Synthesize Deoxyglycosides. Org. Lett..

[20] Luo T., Zhang Y., Xi J., Lu Y., Dong H. (2020). Improved Synthesis of Sulfur-Containing Glycosides by Suppressing Thioacetyl Migration. Front. Chem..

[21] Luo T., Zhang Q., Guo Y.-F., Pei Z.-C., Dong H. (2022). Efficient Preparation of 2-SAc-Glycosyl Donors and Investigation of Their Application in the Synthesis of 2-Deoxyglycosides. Eur. J. Org. Chem..

[22] Luo T., Guo Y.-F., Xu T.-T., Dong H. (2023). Visible-Light-Promoted Desulfurization to Synthesize Deoxyglycosides. J. Org. Chem..

[23] Heuckendorff M., Poulsen L.T., Hedberg C., Jensen H.H. (2018). Dissection of the effects that govern thioglucoside and thiomannoside reactivity. Org. Biomol. Chem..

[24] Smith R., Müller-Bunz H., Zhu X. (2016). Investigation of *α*-Thioglycoside Donors: Reactivity Studies toward Configuration-Controlled Orthogonal Activation in One-Pot Systems. Org. Lett..

[25] Crich D., Li M. (2007). Revisiting the Armed-Disarmed Concept: The Importance of Anomeric Configuration in the Activation of *S*-Benzoxazolyl Glycosides. Org. Lett..

[26] Singh Y., Geringer S.A., Demchenko A.V. (2022). Synthesis and Glycosidation of Anomeric Halides: Evolution from Early Studies to Modern Methods of the 21st Century. Chem. Rev..

[27] Hunsen M., Long D.A., D’Ardenne C.R., Smith A.L. (2005). Mild one-pot preparation of glycosyl bromides. Carbohydr. Res..

[28] Wen P., Simmons C.J., Ma Z.-X., Blaszczyk S.A., Balzer P.G., Ye W., Duan X., Wang H.-Y., Yin D., Stevens C.M. (2020). Synthesis of glycosyl chlorides and bromides by chelation assisted activation of picolinic esters under mild neutral conditions. Org. Lett..

[29] Shadrick M., Singh Y., Demchenko A.V. (2020). Stereocontrolled α-galactosylation under cooperative catalysis. J. Org. Chem..

[30] Singh Y., Geringer S.A., Demchenko A.V. (2020). Defining the scope of the acid-catalyzed glycosidation of glycosyl bromides. Chem. Eur. J..

[31] Ren B., Wang M.-Y., Liu J.-Y., Ge J.-T., Dong H. (2015). Enhanced Basicity of Ag_2_O by Coordination to Soft Anions. ChemCatChem..

[32] Chen H.-M., Withers S.G. (2010). Syntheses of p-nitrophenyl 3- and 4-thio-β-D-glycopyranosides. Carbohydr. Res..

[33] Dong H., Pei Z.-C., Ramström O. (2008). Supramolecular activation in triggered cascade inversion. Chem. Commun..

[34] Sanapala S.R., Kulkarni S.S. (2016). Expedient Route To Access Rare Deoxy Amino _L_-Sugar Building Blocks for the Assembly of Bacterial Glycoconjugates. J. Am. Chem. Soc..

[35] Emmadi M., Kulkarni S.S. (2013). Synthesis of orthogonally protected bacterial, rare-sugar and D-glycosamine building blocks. Nat. Protoc..

[36] Emmadi M., Kulkarni S.S. (2013). Orthogonally protected D-galactosamine thioglycoside building blocks via highly regioselective, double serial and double parallel inversions of *β*-D-thiomannoside. Org. Biomol. Chem..

[37] Dong H., Rahm M., Brinck T., Ramstrom O. (2008). Supramolecular control in carbohydrate epimerization: Discovery of a new anion host–guest system. J. Am. Chem. Soc..

[38] Guo Y.-F., Luo T., Feng G.-J., Liu C.-Y., Dong H. (2022). Efficient Synthesis of 2-OH Thioglycosides from Glycals Based on the Reduction of Aryl Disulfides by NaBH_4_. Molecules.

[39] Cheshev P., Marra A., Dondoni A. (2006). Direct epoxidation of D-glucal and D-galactal derivatives with in situ generated DMDO. Carbohydr. Res..

[40] Marzabadi C.H., Spilling C.D. (1993). Stereoselective Glucal Epoxide Formation1. J. Org. Chem..

[41] Hsu M.Y., Liu Y.P., Lam S., Lin S.C., Wang C.C. (2016). TMSBr mediated solvent and work up free synthesis of α-2-deoxyglycosides from glycals. Beilstein J. Org. Chem..

[42] Yang G.-F., Luo X.-S., Guo H., Wang Q.-B., Zhou J.-F., Huang T.-Y., Tang J., Shan J.-J., Zhang J.-B. (2018). *α*-selective synthesis of 2-deoxy-glycosides and disaccharides. J. Carbohydr. Chem..

[43] Dong H., Pei Z., Angelin M., Byströ S., Ramström O. (2007). Efficient synthesis of β-D-mannosides and β-D-talosides by double parallel or double serial inversion. J. Org. Chem..

