# Supplementary material for: Synthesis of 2-Deoxyglycosides with Exclusive β-Configuration Using 2-SAc Glycosyl Bromide Donors
Source: Molecules. 2025 Jan 5;30(1):185. doi: 10.3390/molecules30010185 (PMC11721102; doi:10.3390/molecules30010185)
Supplement: Supplementary file 1 [file molecules-30-00185-s001.zip › molecules-3384766-supplementary.pdf]

Supplementary Material

Synthesis of 2-Deoxyglycosides with Exclusive β-Configuration Using 2-SAc Glycosyl Bromide Donors

Yang-Fan Guo, Tian-Tian Xu, Guo-Hui Zhang, and Hai Dong*

[a] Key laboratory of Material Chemistry for Energy Conversion and Storage, Ministry of Education, School of Chemistry & Chemical Engineering, Huazhong University of Science & Technology, Luoyu Road 1037, Wuhan, 430074, P. R. China. E-mail: hdong@mail.hust.edu.cn

[b] Hubei Key Laboratory of Bioinorganic Chemistry and Materia Medica,, Huazhong University of Science & Technology, Wuhan, PR China.

TABLE OF CONTENTS

1. ^{1}H and ^{13}C NMR spectra of unknown compounds.... .... S2-S28

Copy of NMR Spectra

Methyl 2,3,4-tri-*O*-benzoyl-6-*O*-(2-*S*-acetyl-3,4,6-tri-*O*-acetyl-2-thio-*β*-D-glucopyranoside)-*α*-D-glucopyranoside 2a

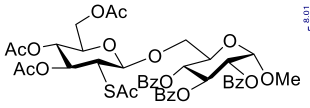

2a

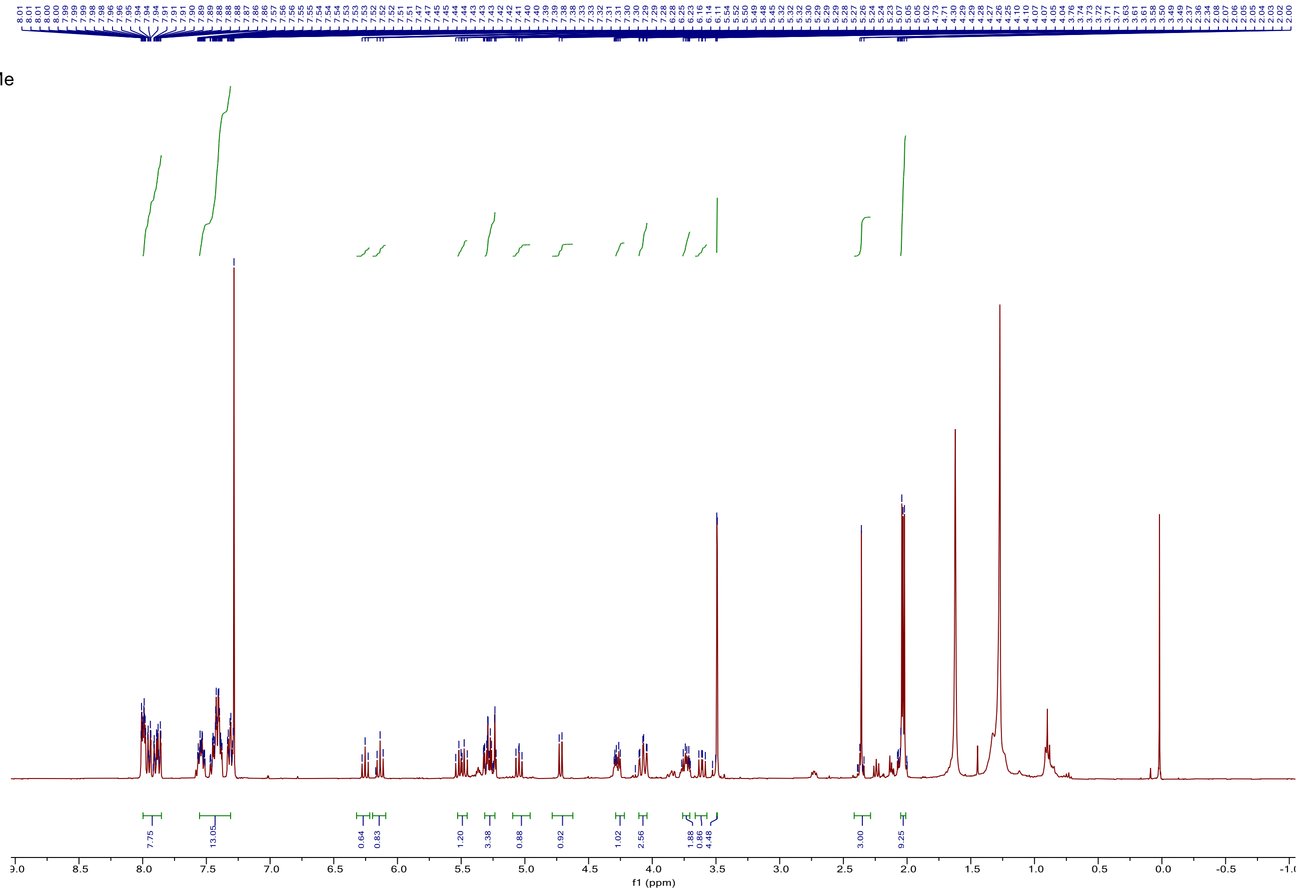

Figure S1. ^{1}H NMR spectrum (400 MHz) of **2a** in $CDCl_3$

Methyl 2,3,4-tri-*O*-benzoyl-6-*O*-(2-*S*-acetyl-3,4,6-tri-*O*-acetyl-2-thio-*β*-D-glucopyranoside)-*α*-D-glucopyranoside 2a

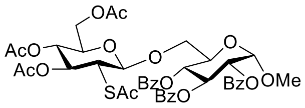

2a

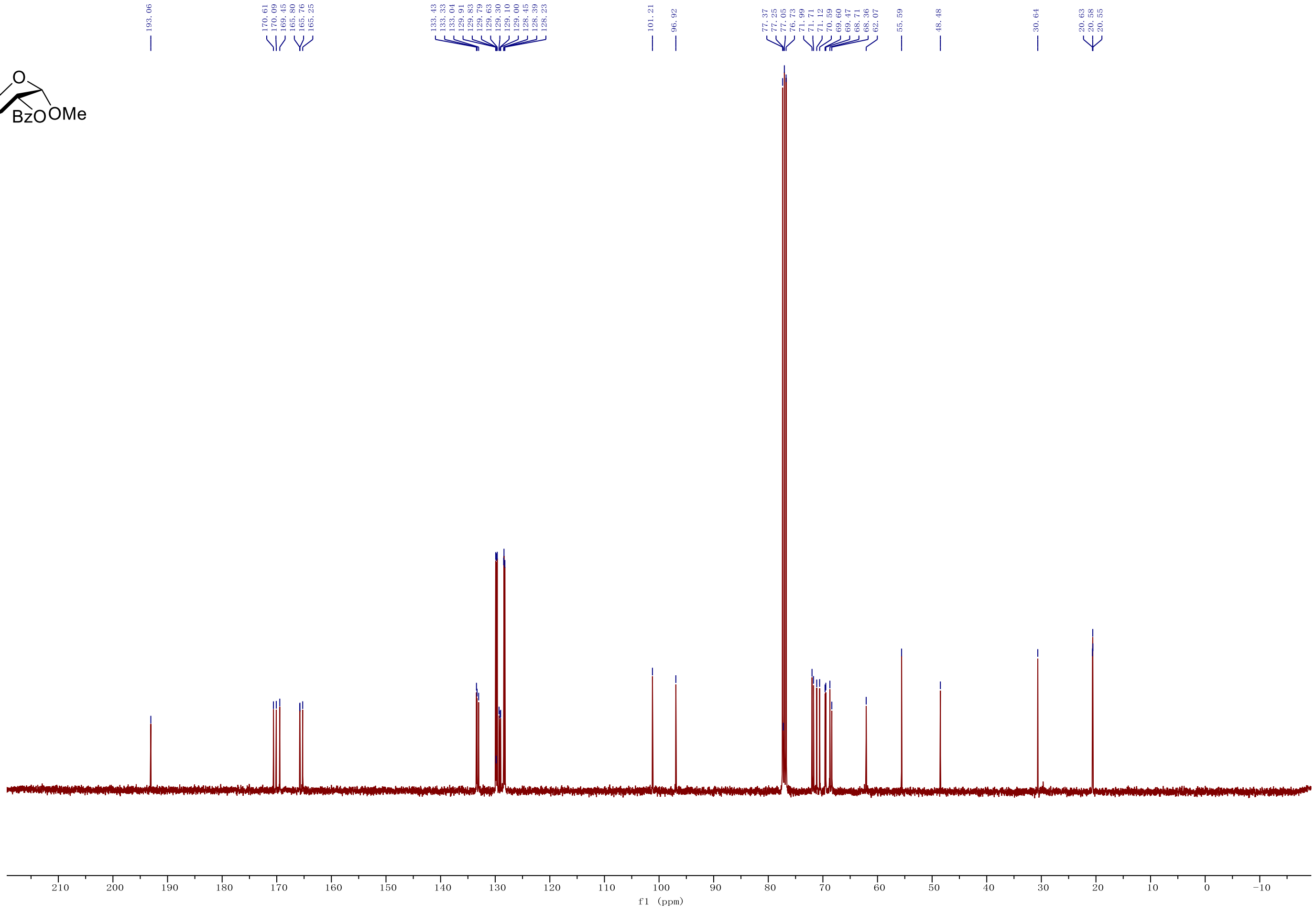

Figure S2. ^{13}C NMR spectrum (101 MHz) of **2a** in $CDCl_3$

Methyl 2,3,4-tri-*O*-benzyl-6-*O*-(2-*S*-acetyl-3,4,6-tri-*O*-acetyl-2-thio-*β*-D-glucopyranoside)-*α*-D-glucopyranoside 2b

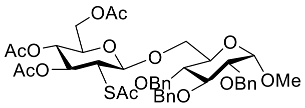

2b

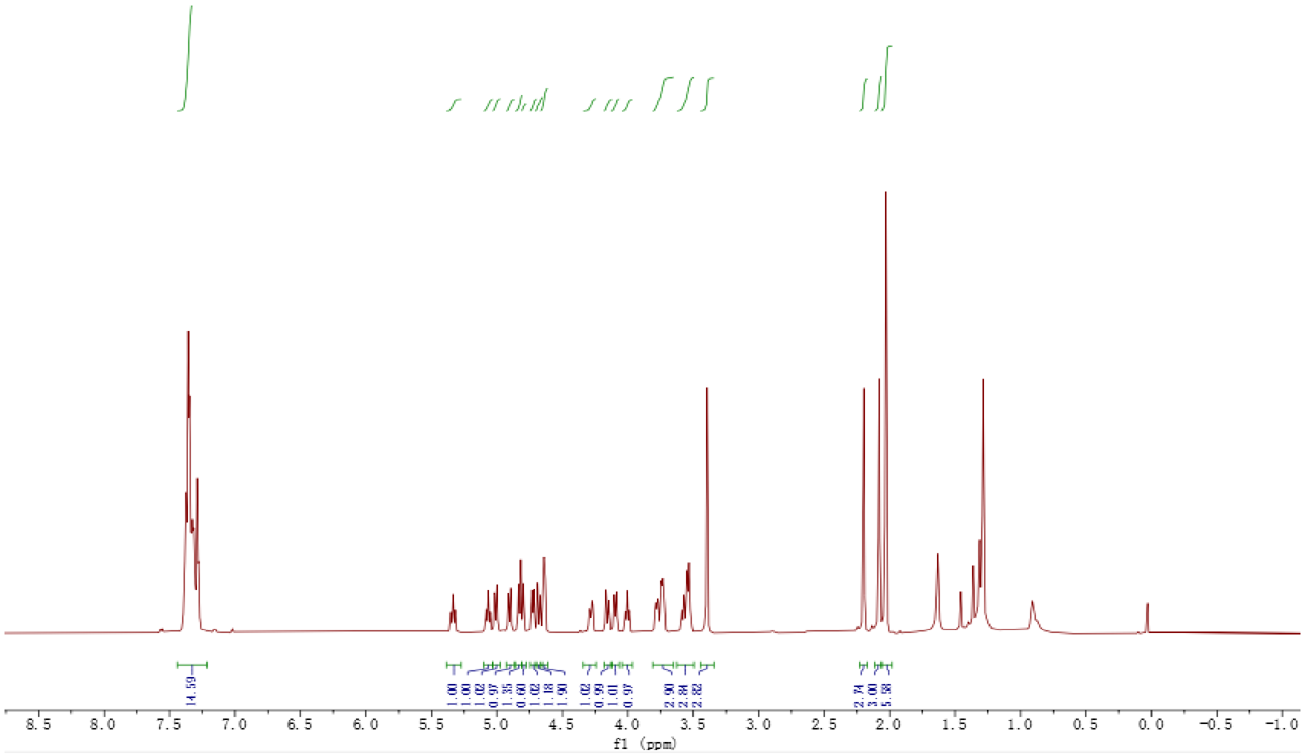

Figure S3. ^{1}H NMR spectrum (600 MHz) of **2b** in $CDCl_3$

Methyl 2,3,4-tri-*O*-benzyl-6-*O*-(2-*S*-acetyl-3,4,6-tri-*O*-acetyl-2-thio-*β*-D-glucopyranoside)-*α*-D-glucopyranoside 2b

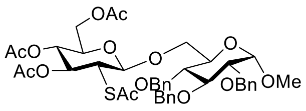

2b

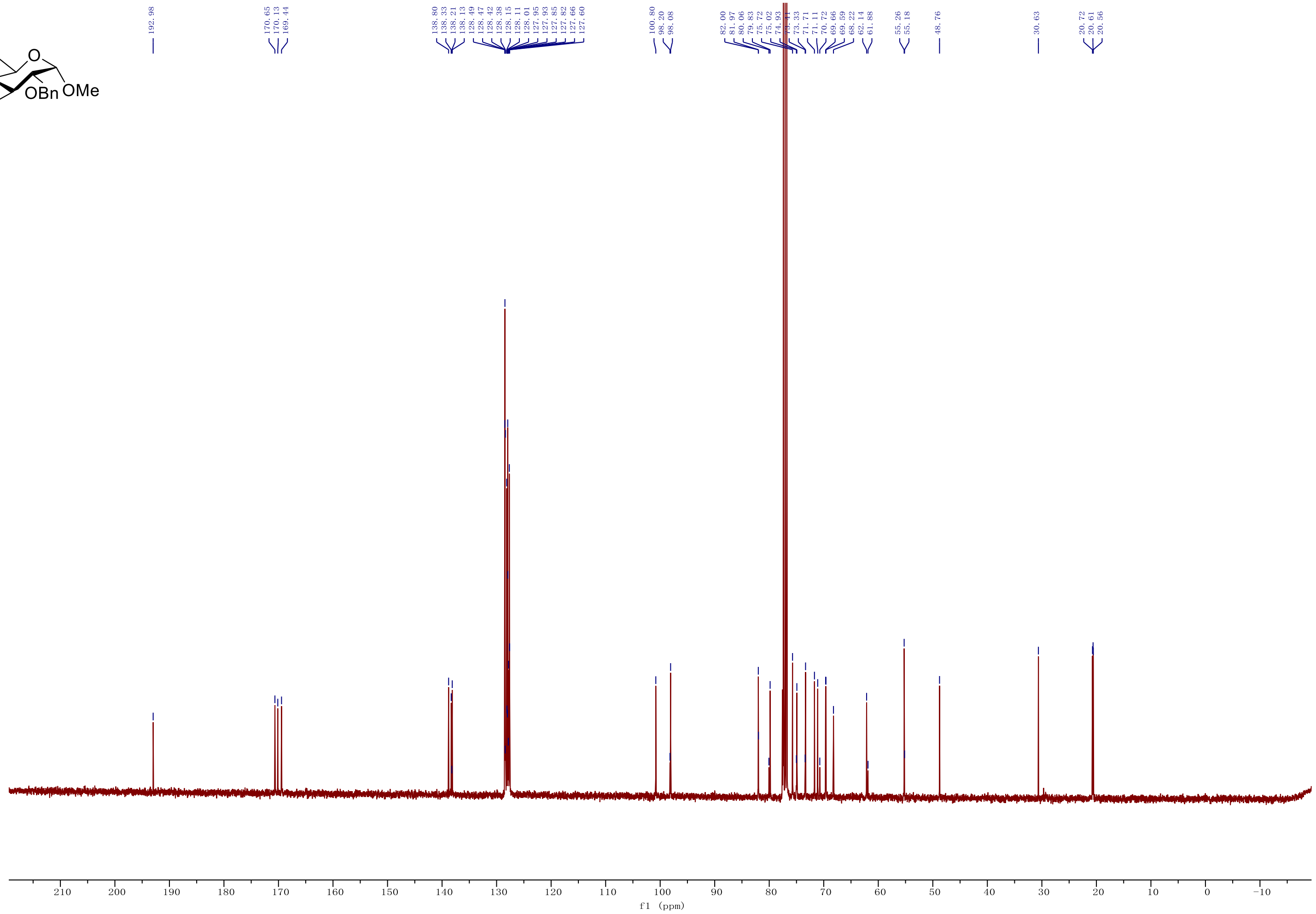

Figure S4. ^{13}C NMR spectrum (101 MHz) of **2b** in $CDCl_3$

Methyl 2,3,6-tri-*O*-benzyl-4-*O*-(2-*S*-acetyl-3,4,6-tri-*O*-acetyl-2-thio-*β*-D-glucopyranoside)-*α*-D-glucopyranoside 2c

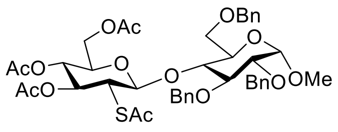

2c

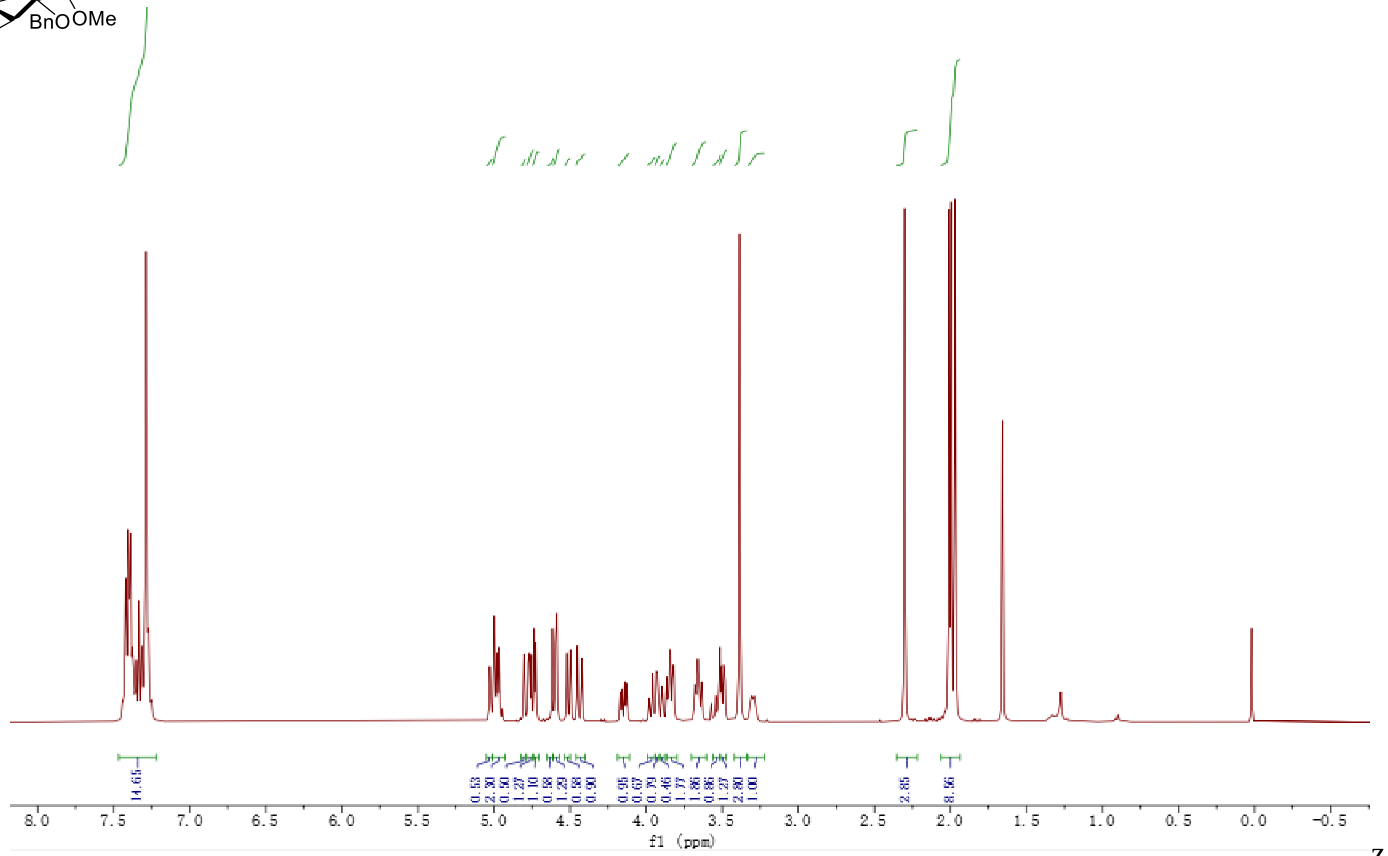

Z

Figure S5. ^{1}H NMR spectrum (400 MHz) of **2c** in $CDCl_3$

Methyl 2,3,6-tri-*O*-benzyl-4-*O*-(2-*S*-acetyl-3,4,6-tri-*O*-acetyl-2-thio-*β*-D-glucopyranoside)-*α*-D-glucopyranoside 2c

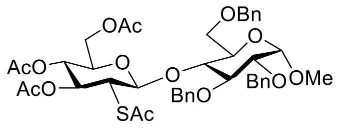

2c

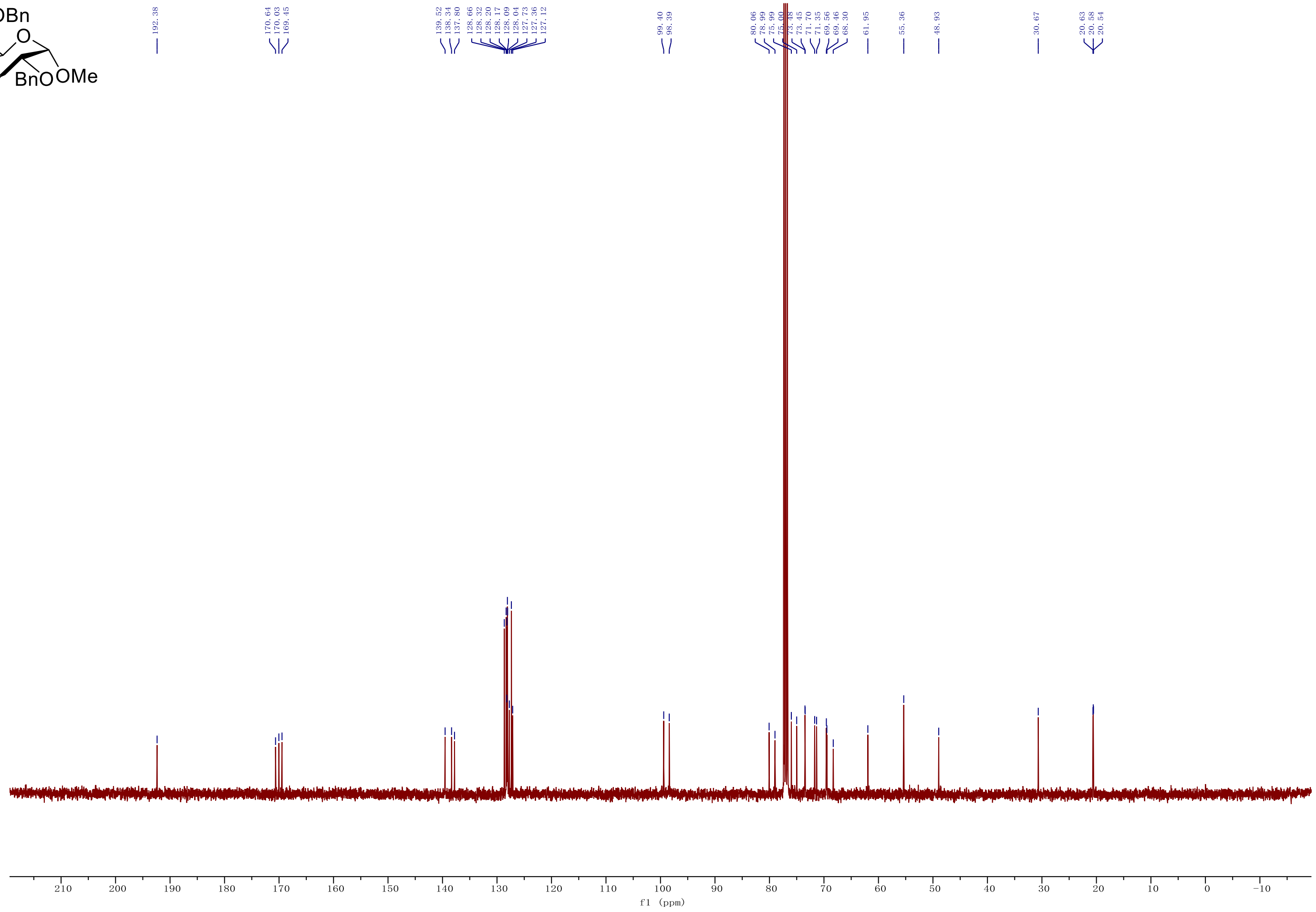

Figure S6. ^{13}C NMR spectrum (101 MHz) of **2c** in $CDCl_3$

Methyl 2-*O*-benzoyl-4,6-*O*-benzylidene-3-*O*-(2-*S*-acetyl-3,4,6-tri-*O*-acetyl-2-thio-*β*-D-glucopyranoside)-*α*-D-glucopyranoside 2e

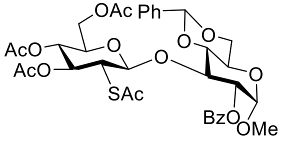

2e

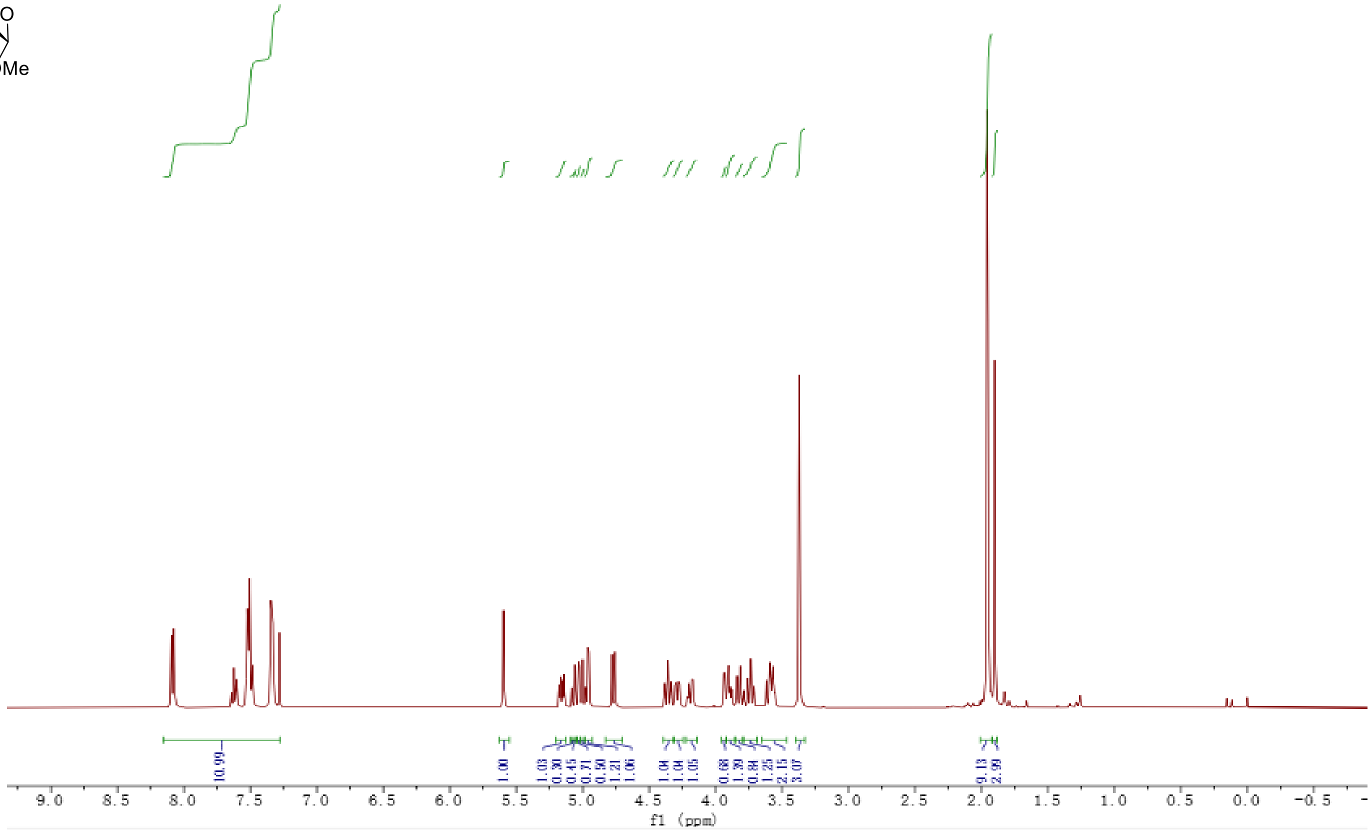

Figure S7. ^{1}H NMR spectrum (400 MHz) of **2e** in $CDCl_3$

Methyl 2-*O*-benzoyl-4,6-*O*-benzylidene-3-*O*-(2-*S*-acetyl-3,4,6-tri-*O*-acetyl-2-thio-*β*-D-glucopyranoside)-*α*-D-glucopyranoside 2e

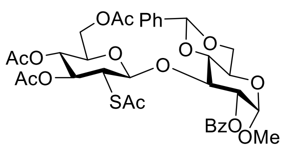

2e

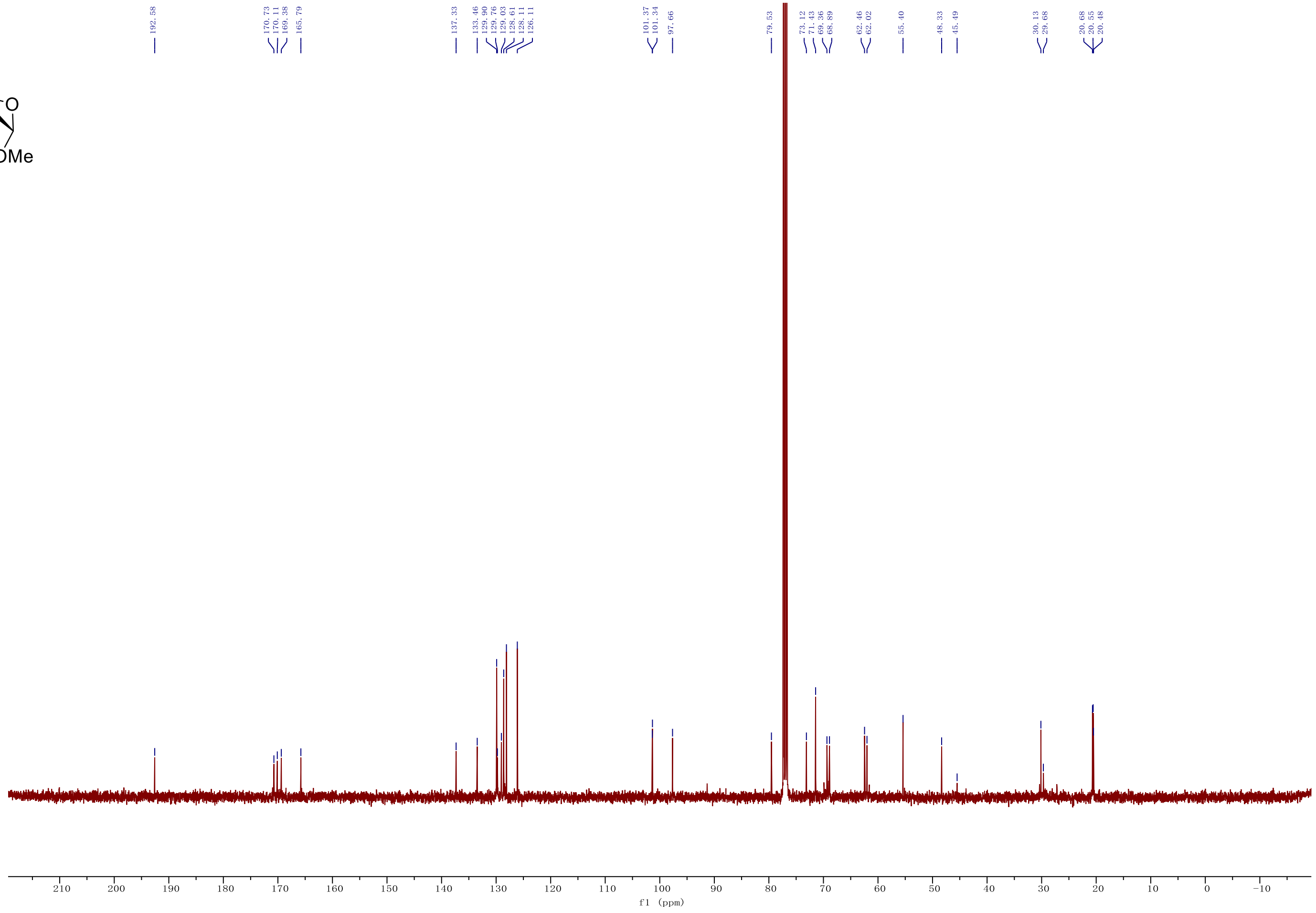

Figure S8. ^{13}C NMR spectrum (101 MHz) of **2e** in $CDCl_3$

Methyl 2-*O*-benzoyl-3,4,6-*O*-benzoyl-2-*O*-(2-*S*-acetyl-3,4,6-tri-*O*-acetyl-2-thio-*β*-D-glucopyranoside)-*β*-D-glucopyranoside 2f

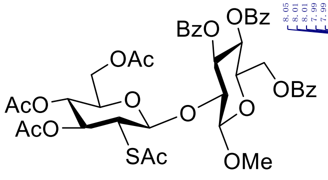

2f

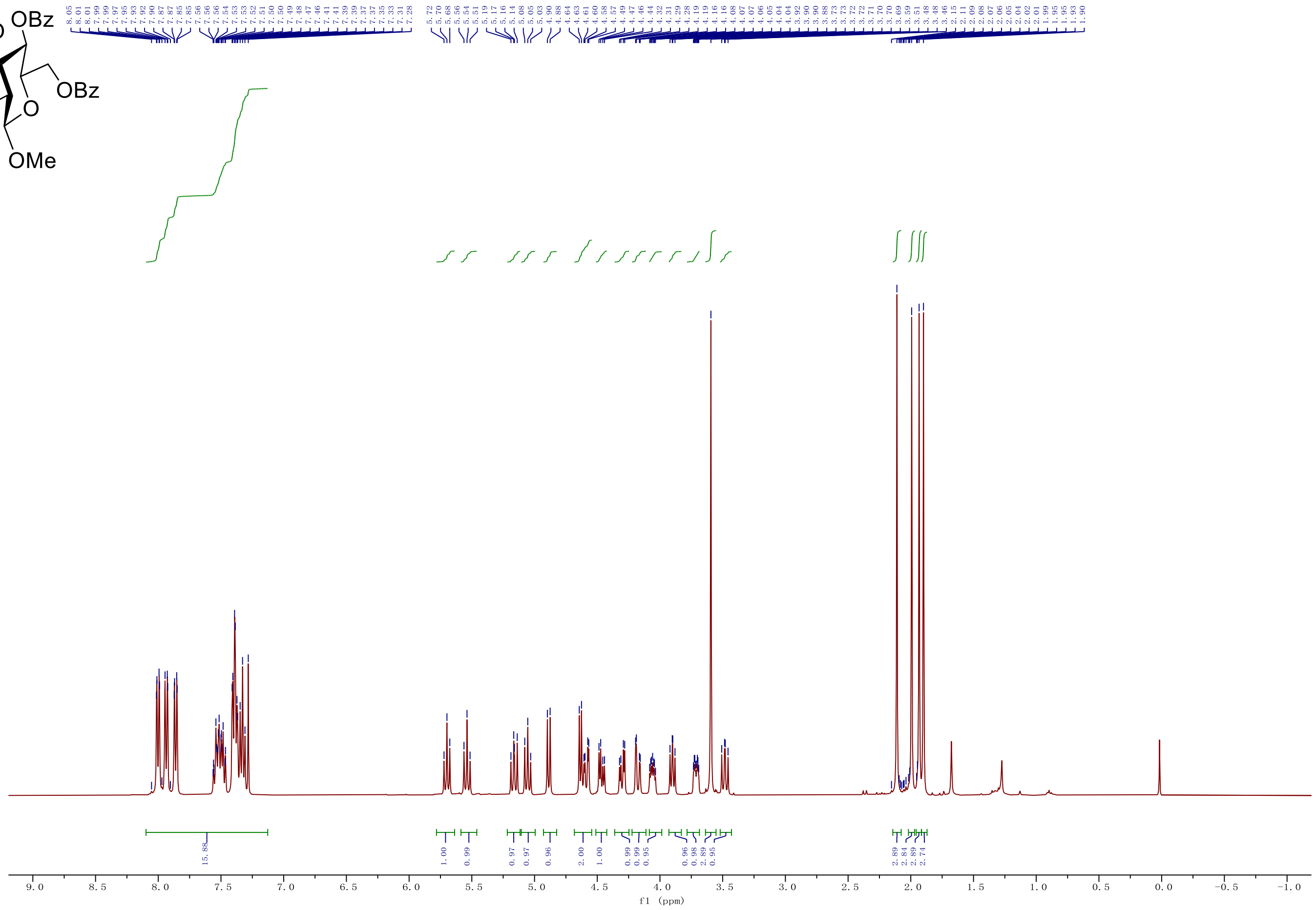

Figure S9. ^{1}H NMR spectrum (400 MHz) of **2f** in $CDCl_3$

Methyl 2-*O*-benzoyl-3,4,6-*O*-benzoyl-2-*O*-(2-*S*-acetyl-3,4,6-tri-*O*-acetyl-2-thio-*β*-D-glucopyranoside)-*β*-D-glucopyranoside 2f

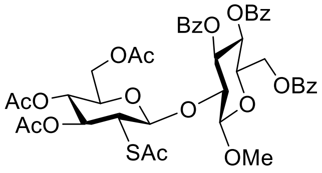

2f

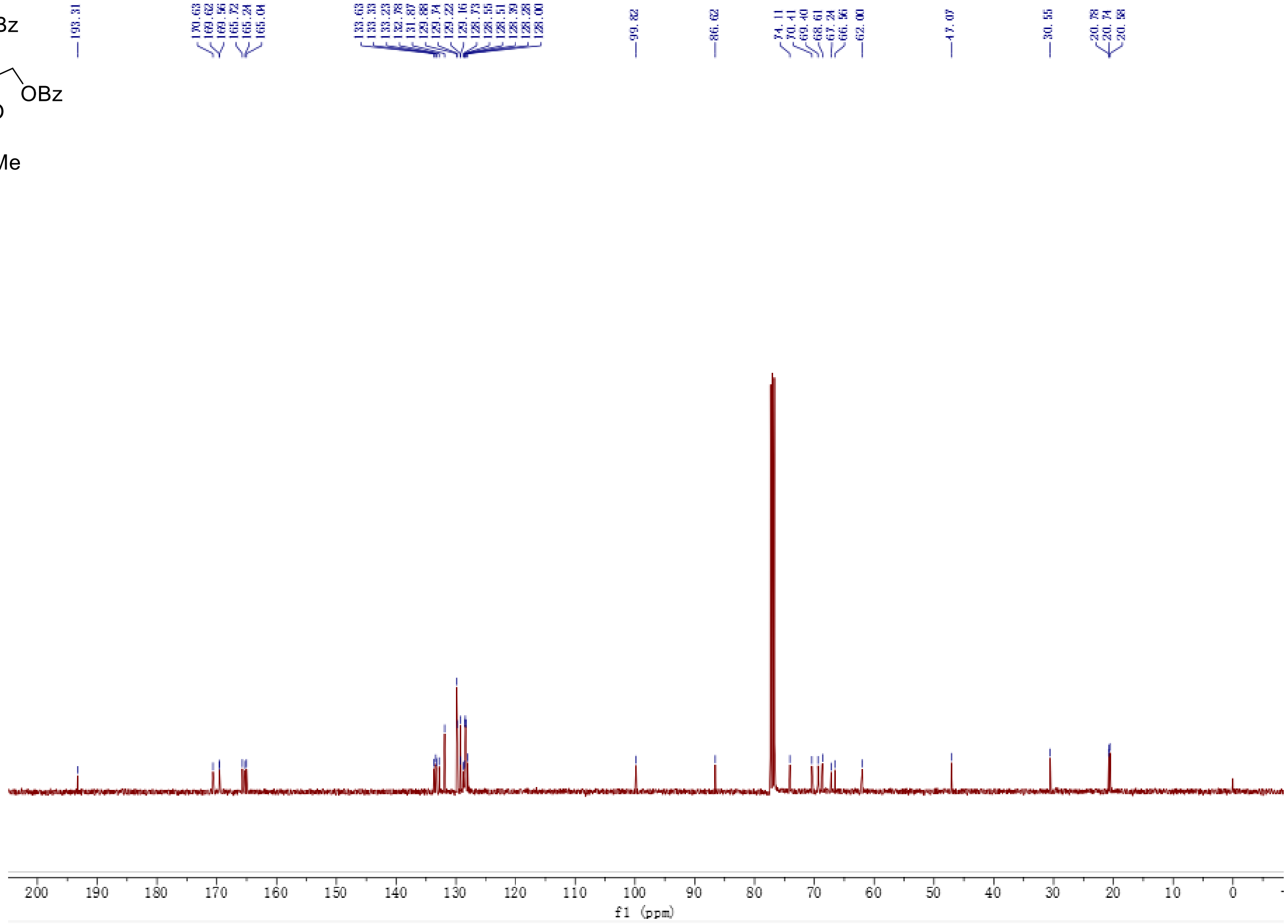

Figure S10. ^{13}C NMR spectrum (101 MHz) of **2f** in $CDCl_3$

Phenyl 2-*S*-acetyl-3,4,6-tri-*O*-acetyl-2-thio-*β*-D-glucopyranoside 2g

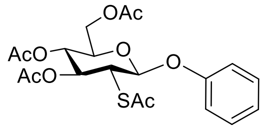

2g

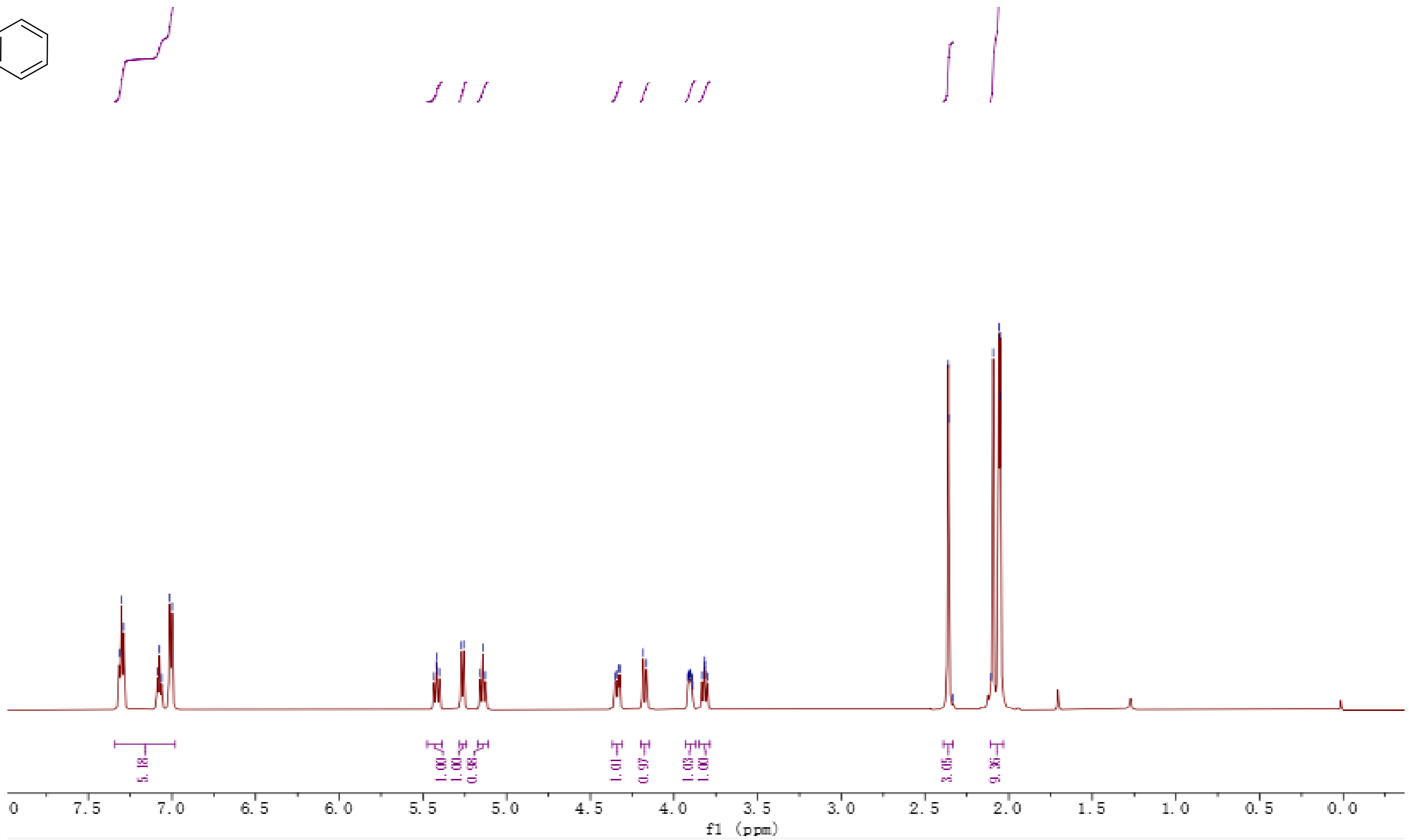

Figure S11. ^{1}H NMR spectrum (600 MHz) of **2g** in $CDCl_3$

Phenyl 2-*S*-acetyl-3,4,6-tri-*O*-acetyl-2-thio-*β*-D-glucopyranoside 2g

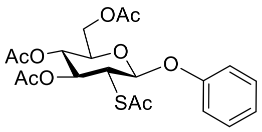

2g

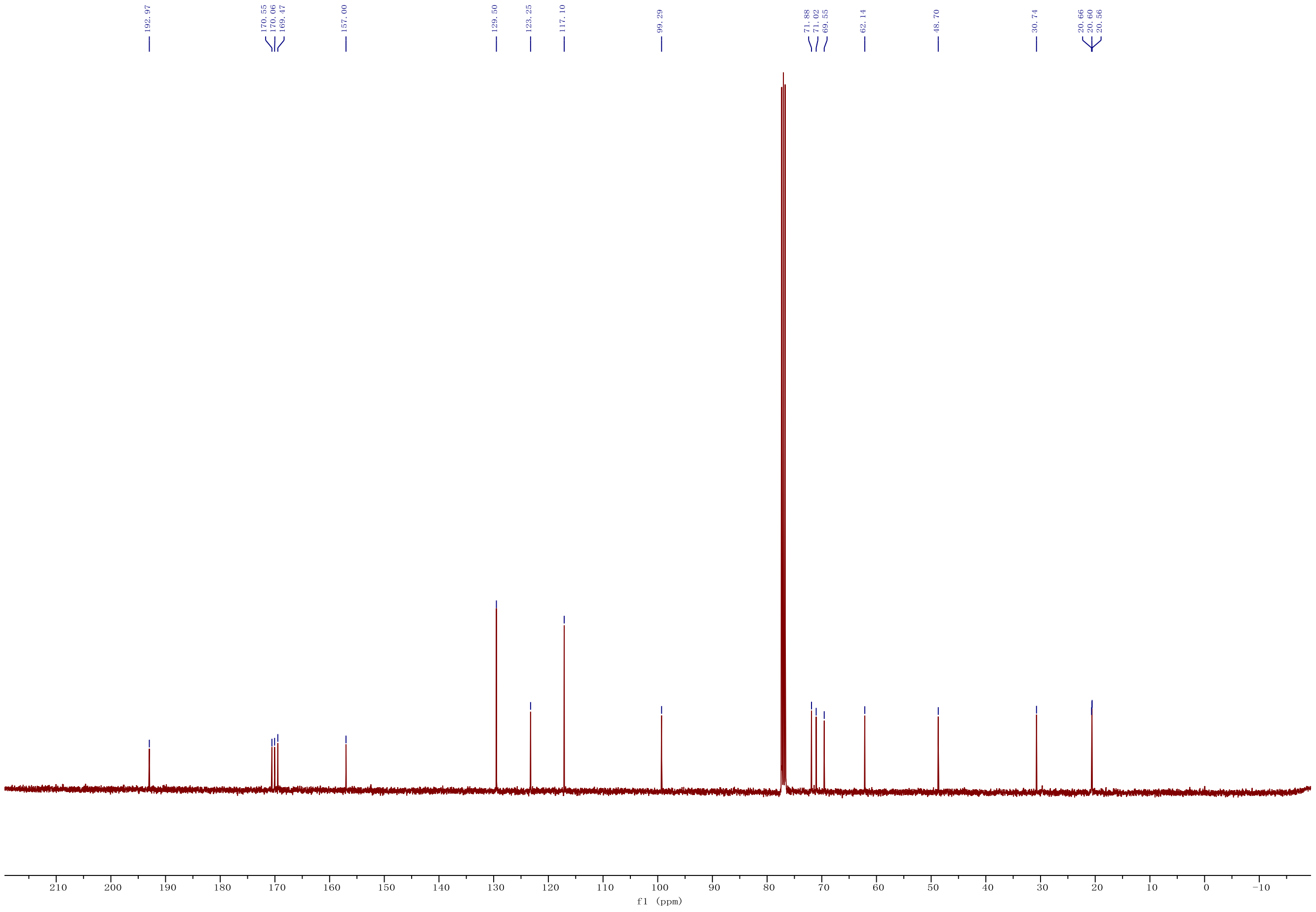

Figure S12. ^{13}C NMR spectrum (101 MHz) of **2g** in $CDCl_3$

p-methoxyphenol 2-*S*-acetyl-3,4,6-tri-*O*-acetyl-2-thio-*β*-D-glucopyranoside 2i

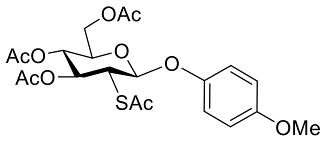

2i

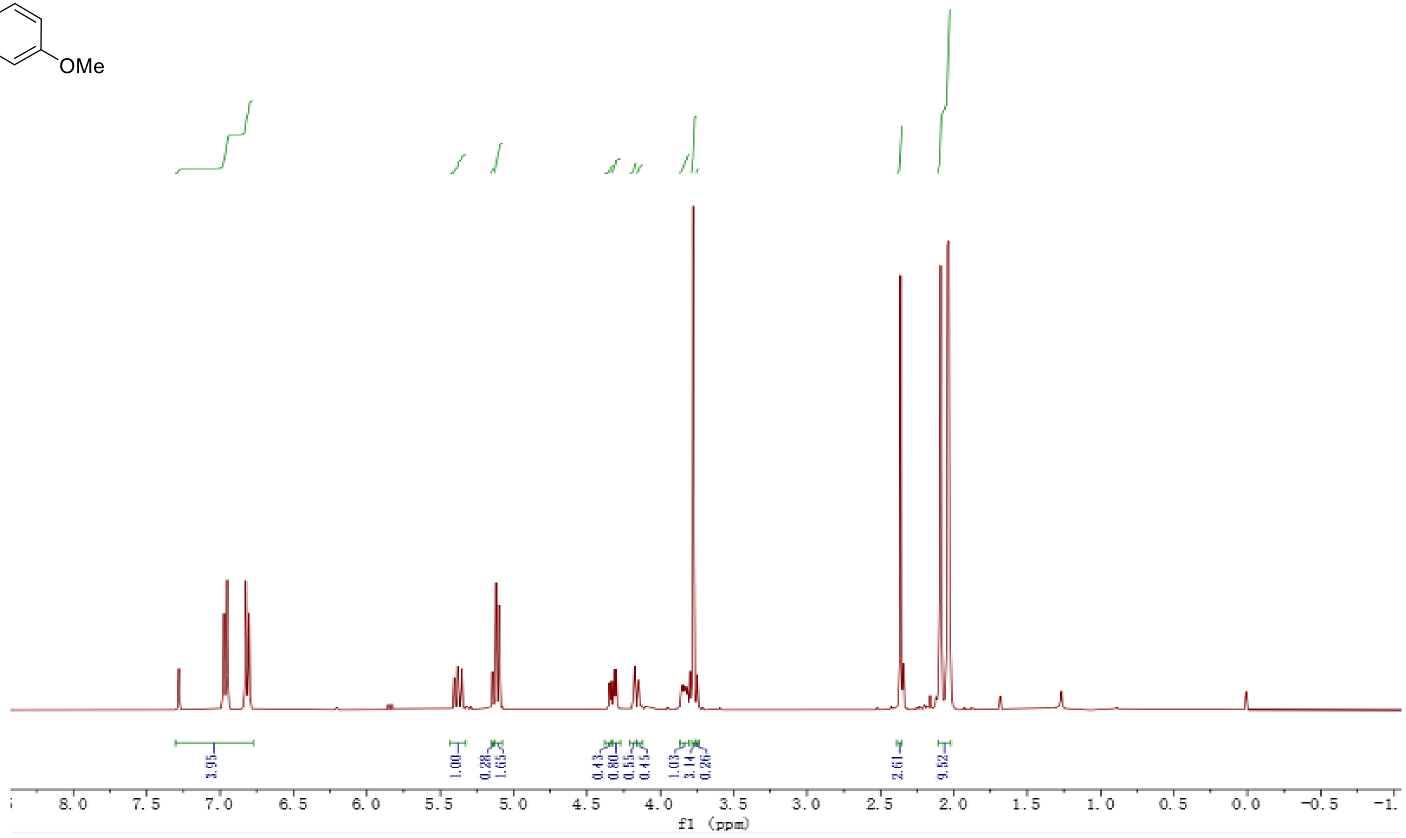

Figure S13. ^{1}H NMR spectrum (400 MHz) of **2i** in $CDCl_3$

***p*-methoxyphenol 2-*S*-acetyl-3,4,6-tri-*O*-acetyl-2-thio-*β*-D-glucopyranoside 2i**

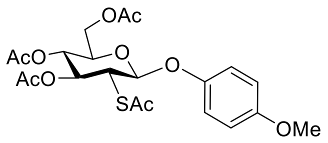

2i

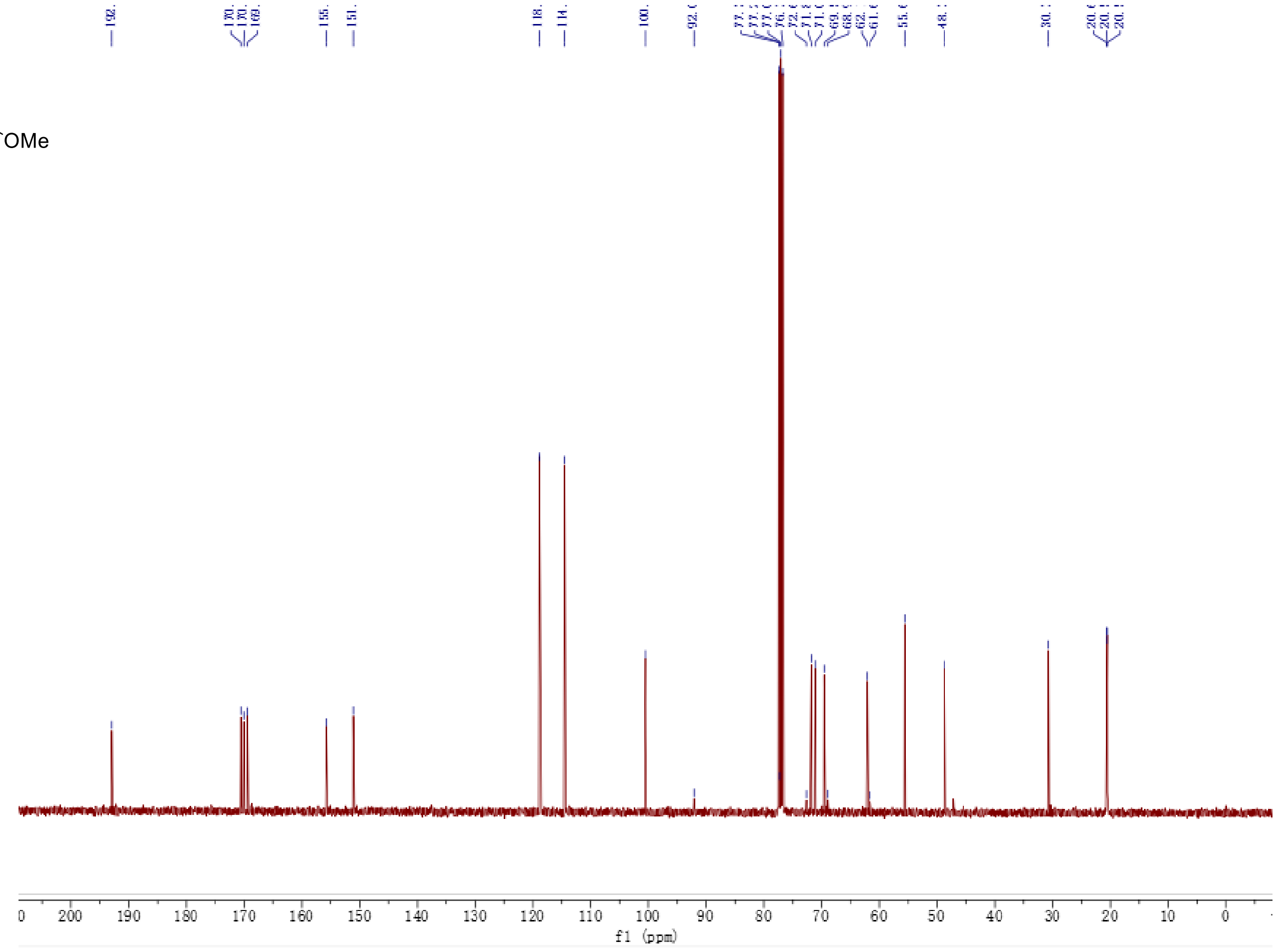

Figure S14. ^{13}C NMR spectrum (101 MHz) of **2i** in $CDCl_3$

Methyl 2,3,4-tri-*O*-benzoyl-6-*O*-(2-*S*-acetyl-3,4,6-tri-*O*-benzoyl-2-thio-*β*-D-galactopyranoside)-*α*-D-glucopyranoside 3a

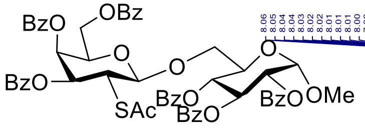

3a

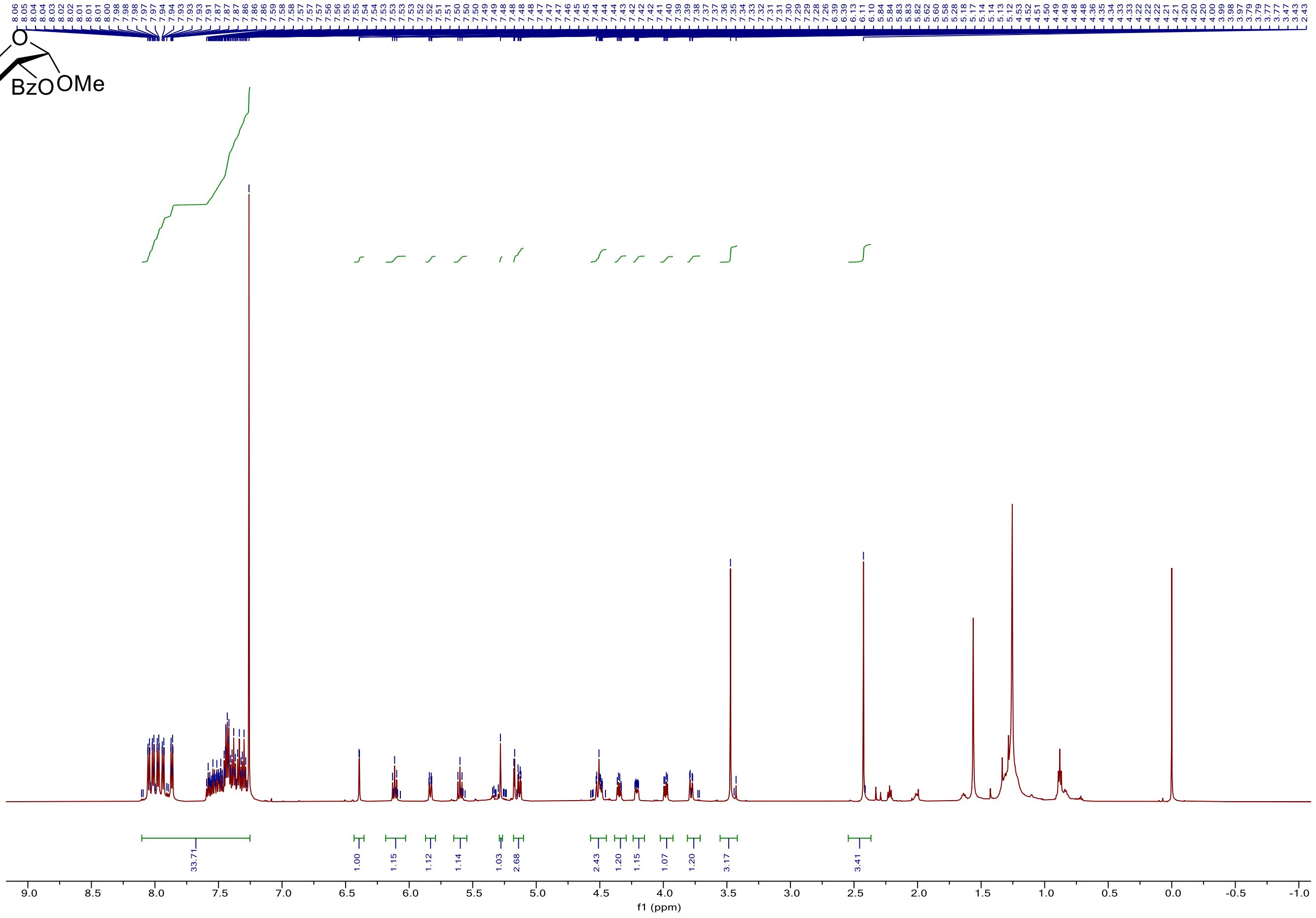

Figure S15. ^{1}H NMR spectrum (600 MHz) of **3a** in $CDCl_3$

Methyl 2,3,4-tri-*O*-benzoyl-6-*O*-(2-*S*-acetyl-3,4,6-tri-*O*-benzoyl-2-thio-*β*-D-galactopyranoside)-*α*-D-glucopyranoside 3a

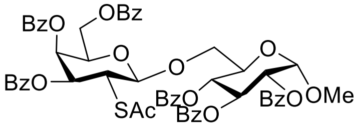

3a

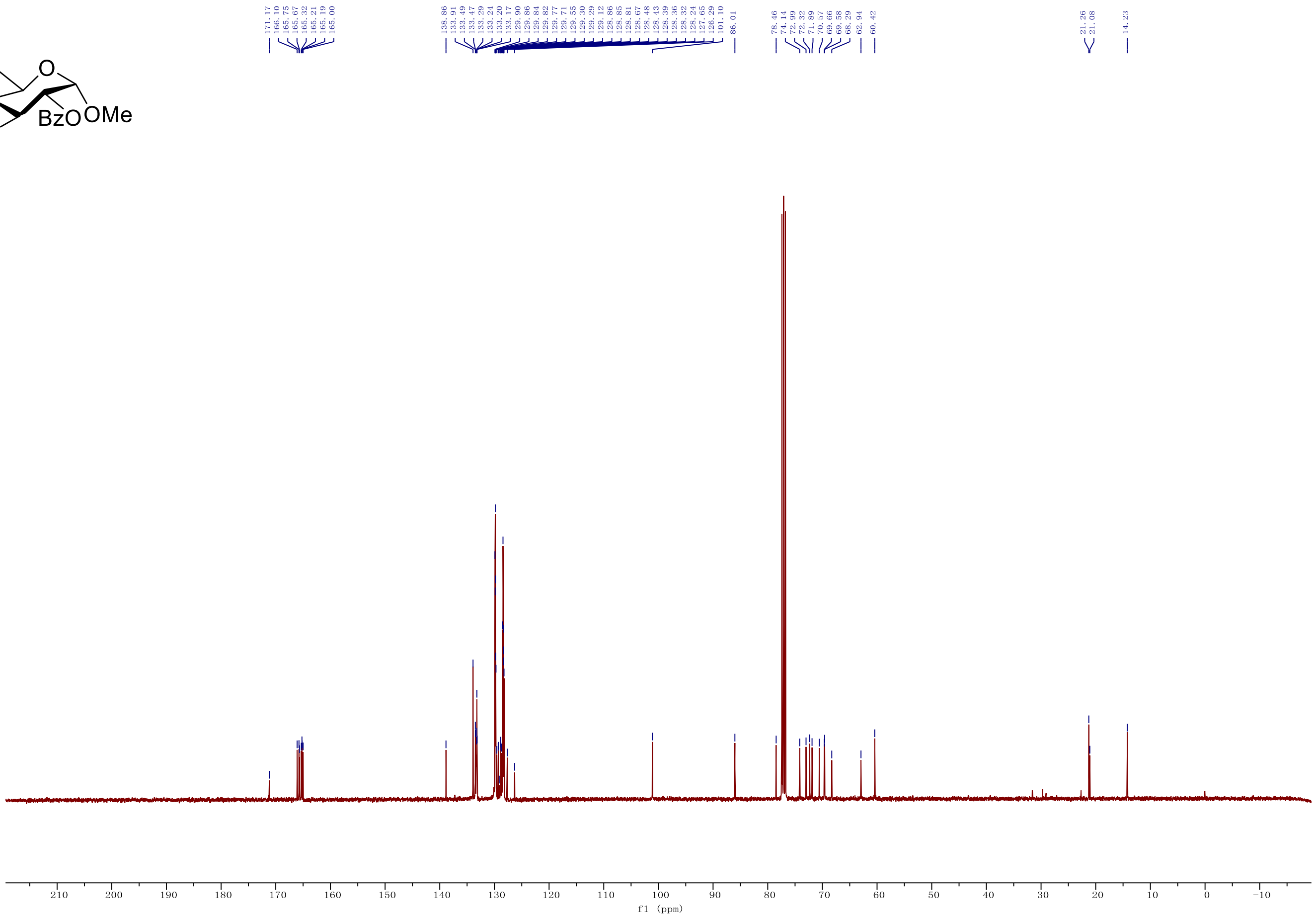

Figure S16. ^{13}C NMR spectrum (101 MHz) of **3a** in $CDCl_3$

Methyl 2,3,4-tri-*O*-benzyl-6-*O*-(2-*S*-acetyl-3,4,6-tri-*O*-benzoyl-2-thio-*β*-D-galactopyranoside)-*α*-D-glucopyranoside 3b

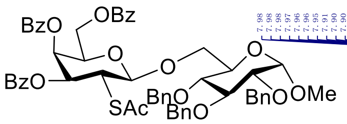

3b

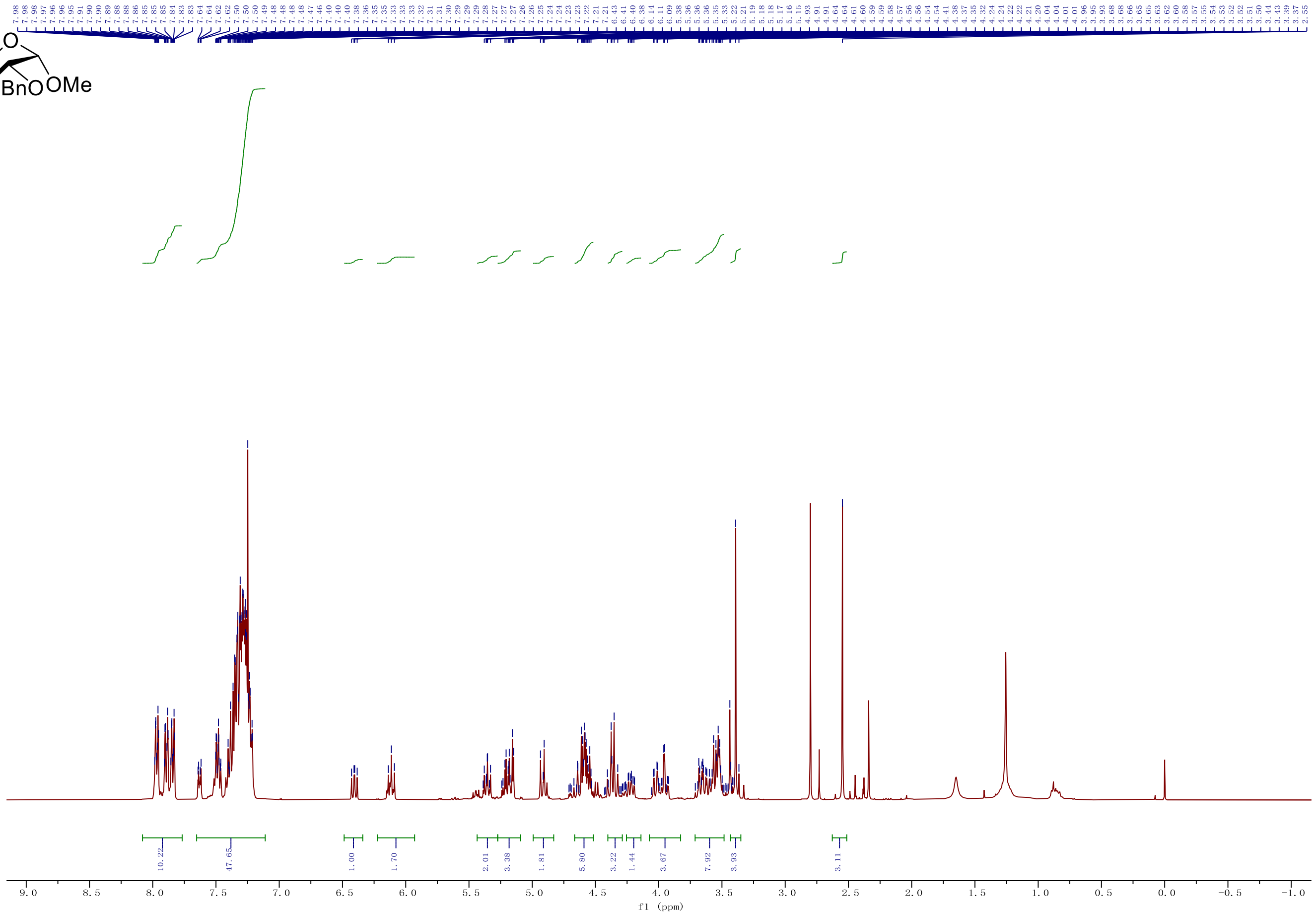

Figure S17. ^{1}H NMR spectrum (400 MHz) of **3b** in $CDCl_3$

Methyl 2,3,4-tri-*O*-benzyl-6-*O*-(2-*S*-acetyl-3,4,6-tri-*O*-benzoyl-2-thio-*β*-D-galactopyranoside)-*α*-D-glucopyranoside 3b

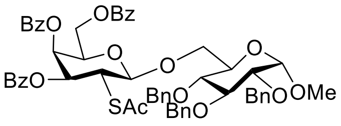

3b

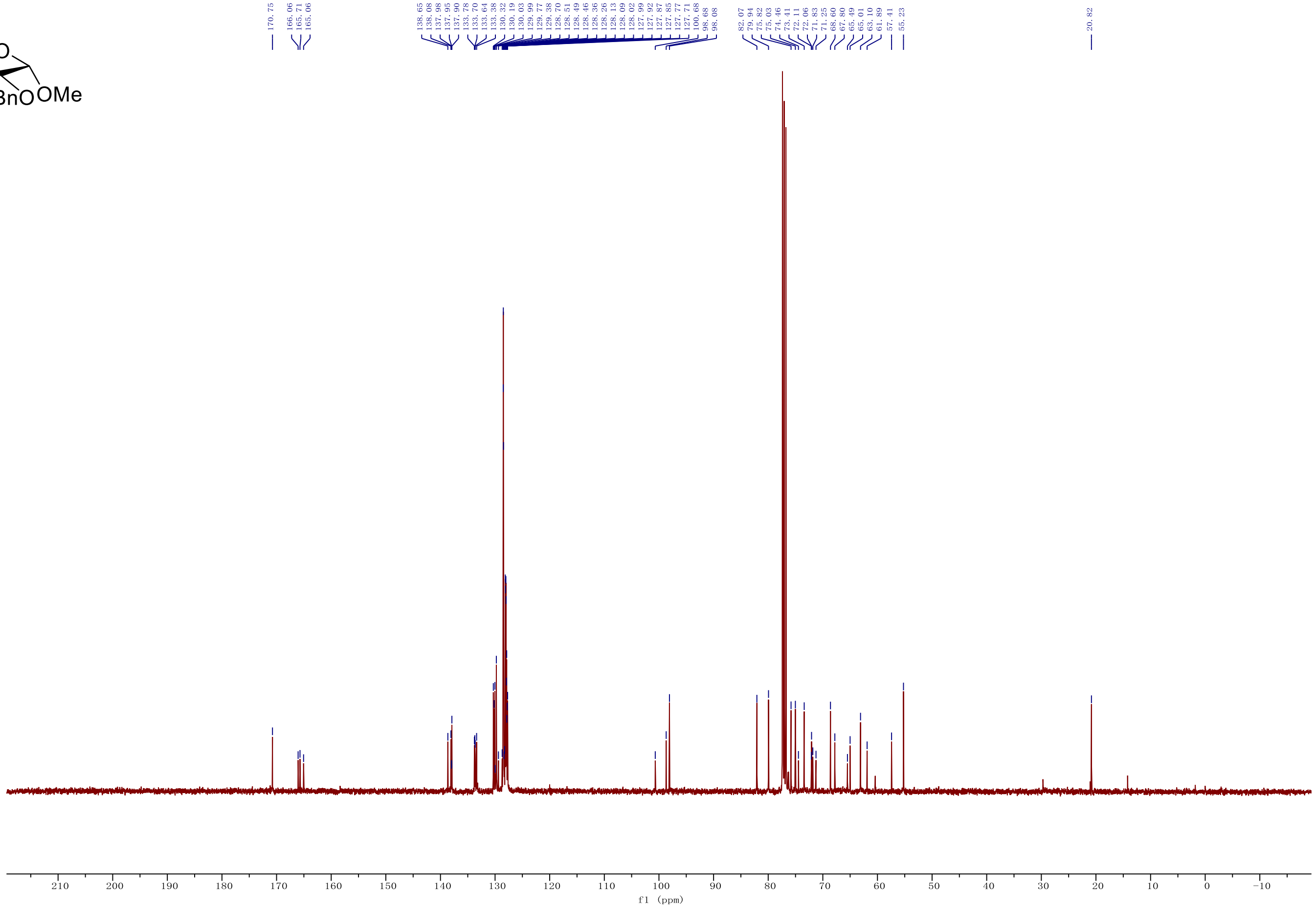

Figure S18. ^{13}C NMR spectrum (101 MHz) of **3b** in $CDCl_3$

Methyl 2,3,6-tri-*O*-benzoyl-4-*O*-(2-*S*-acetyl-3,4,6-tri-*O*-benzoyl-2-thio-*β*-D-galactopyranoside)-*α*-D-glucopyranoside 3d

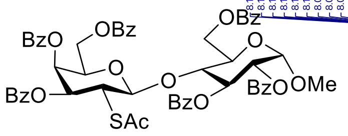

3d

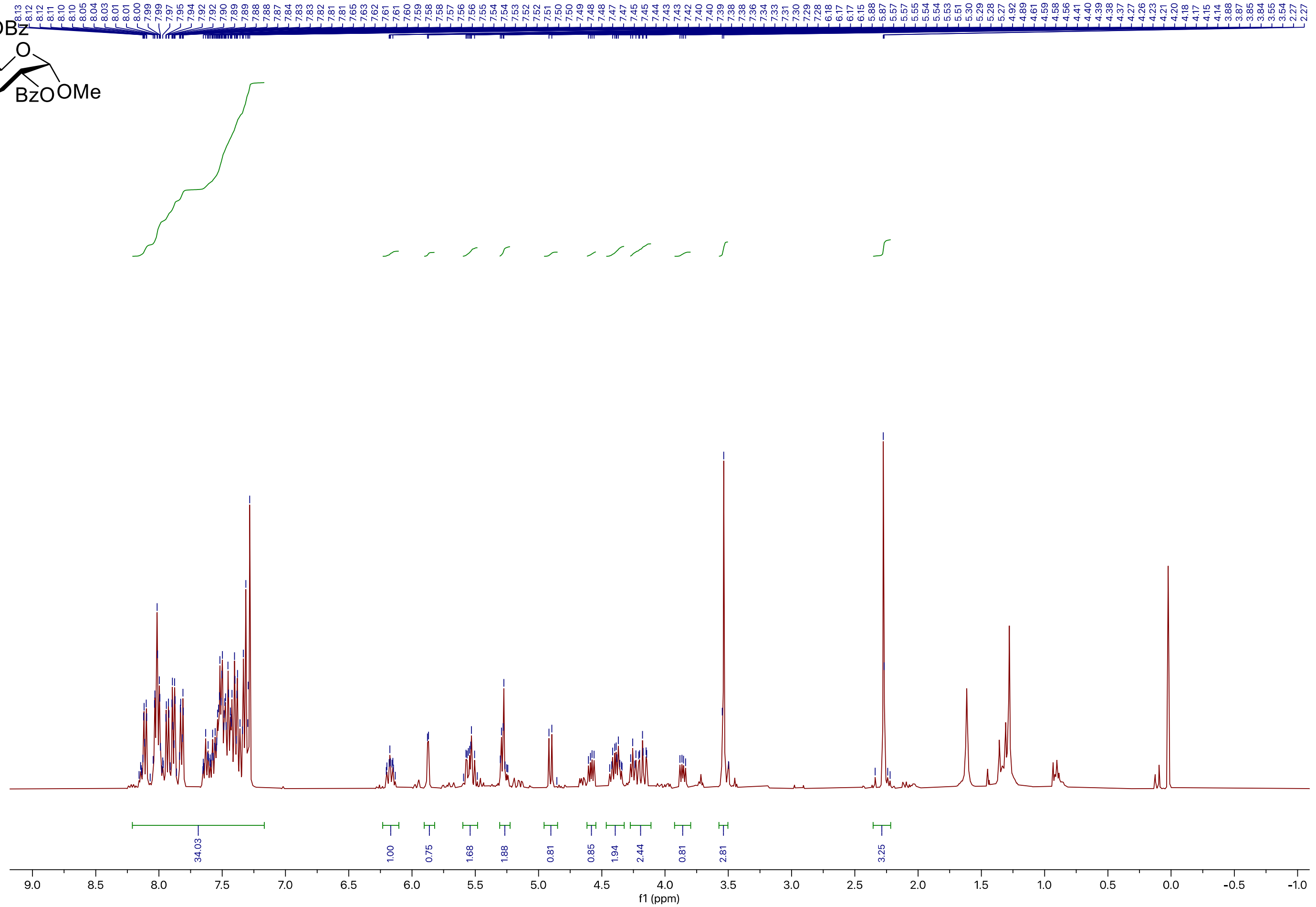

Figure S19. ^{1}H NMR spectrum (400 MHz) of **3d** in $CDCl_3$

Methyl 2,3,6-tri-*O*-benzoyl-4-*O*-(2-*S*-acetyl-3,4,6-tri-*O*-benzoyl-2-thio-*β*-D-galactopyranoside)-*α*-D-glucopyranoside 3d

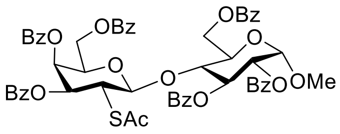

3d

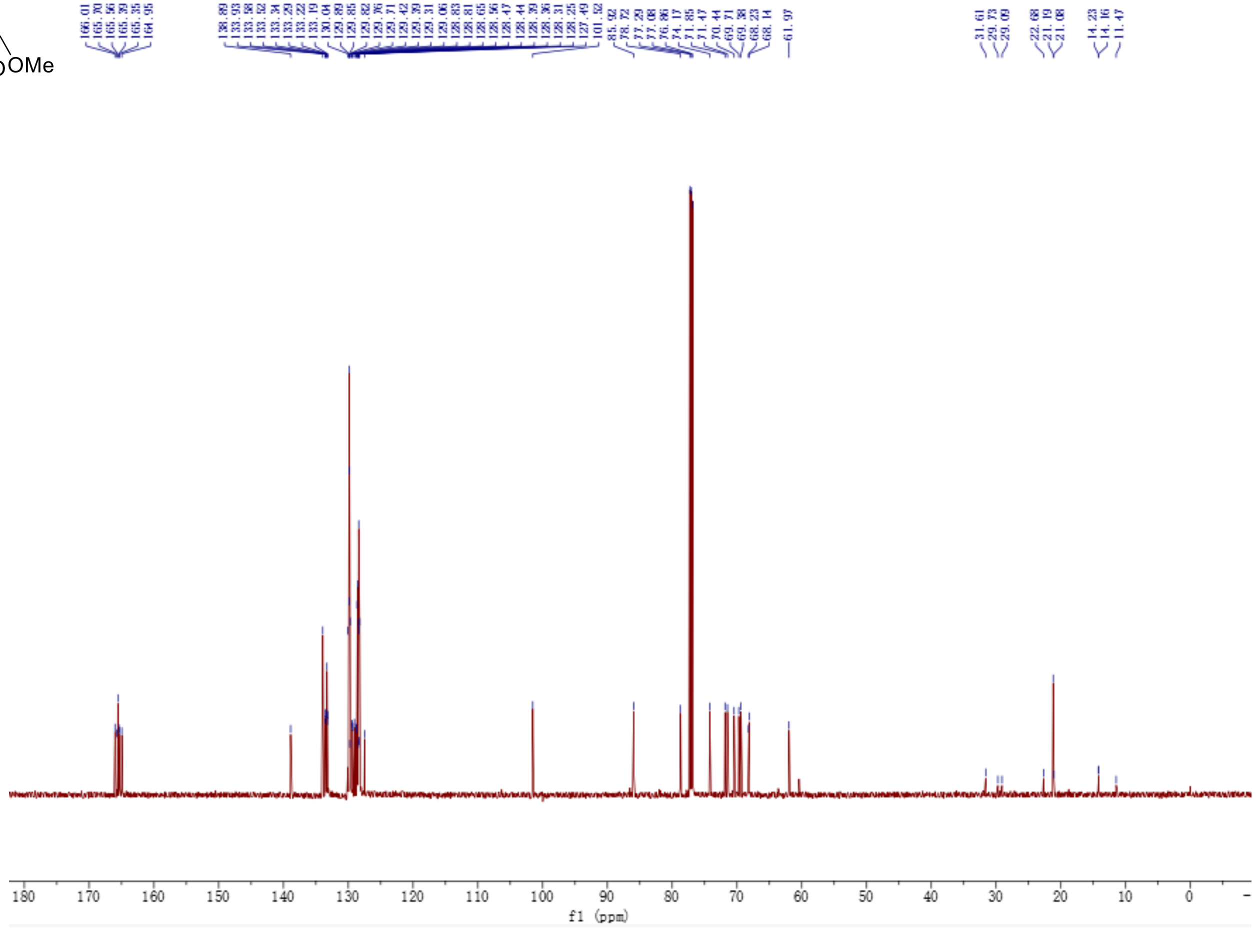

Figure S20. ^{13}C NMR spectrum (151 MHz) of **3d** in $CDCl_3$

Phenyl 2-*S*-acetyl-3,4,6-tri-*O*-acetyl-2-thio-*β*-D-glucopyranoside 3g

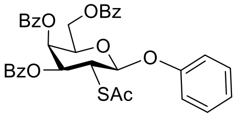

3g

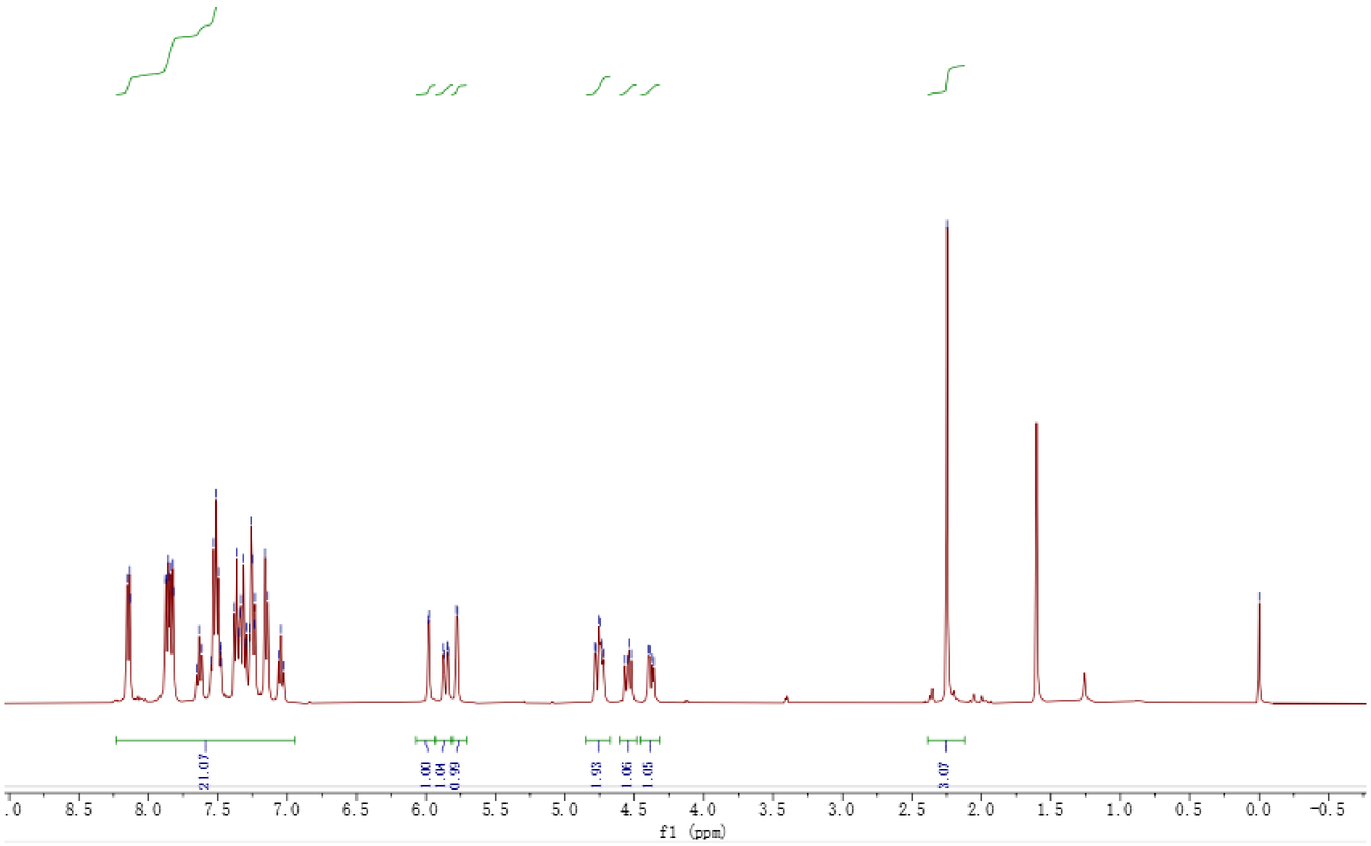

Figure S21. ^{1}H NMR spectrum (600 MHz) of **3g** in $CDCl_3$

Phenyl 2-*S*-acetyl-3,4,6-tri-*O*-acetyl-2-thio-*β*-D-glucopyranoside 3g

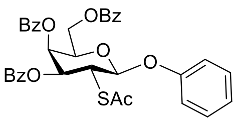

3g

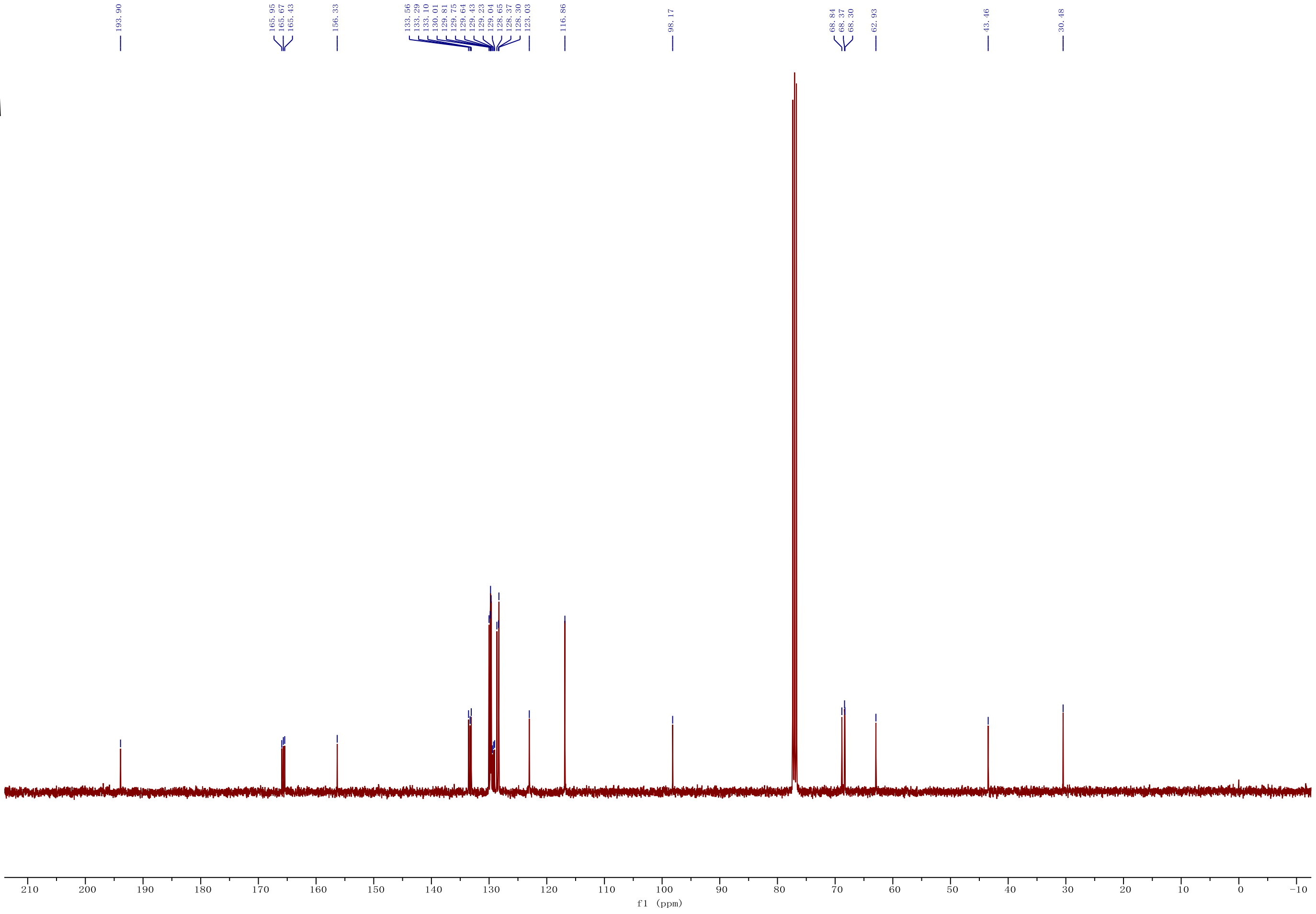

Figure S22. ^{13}C NMR spectrum (101 MHz) of **3g** in $CDCl_3$

Methyl 2,3,4-tri-*O*-benzoyl-6-*O*-(3,4,6-tri-*O*-acetyl-2-deoxy-*β*-D-glucopyranoside)-*α*-D-glucopyranoside 2aD

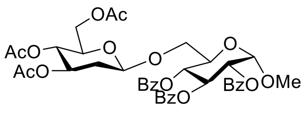

2aD

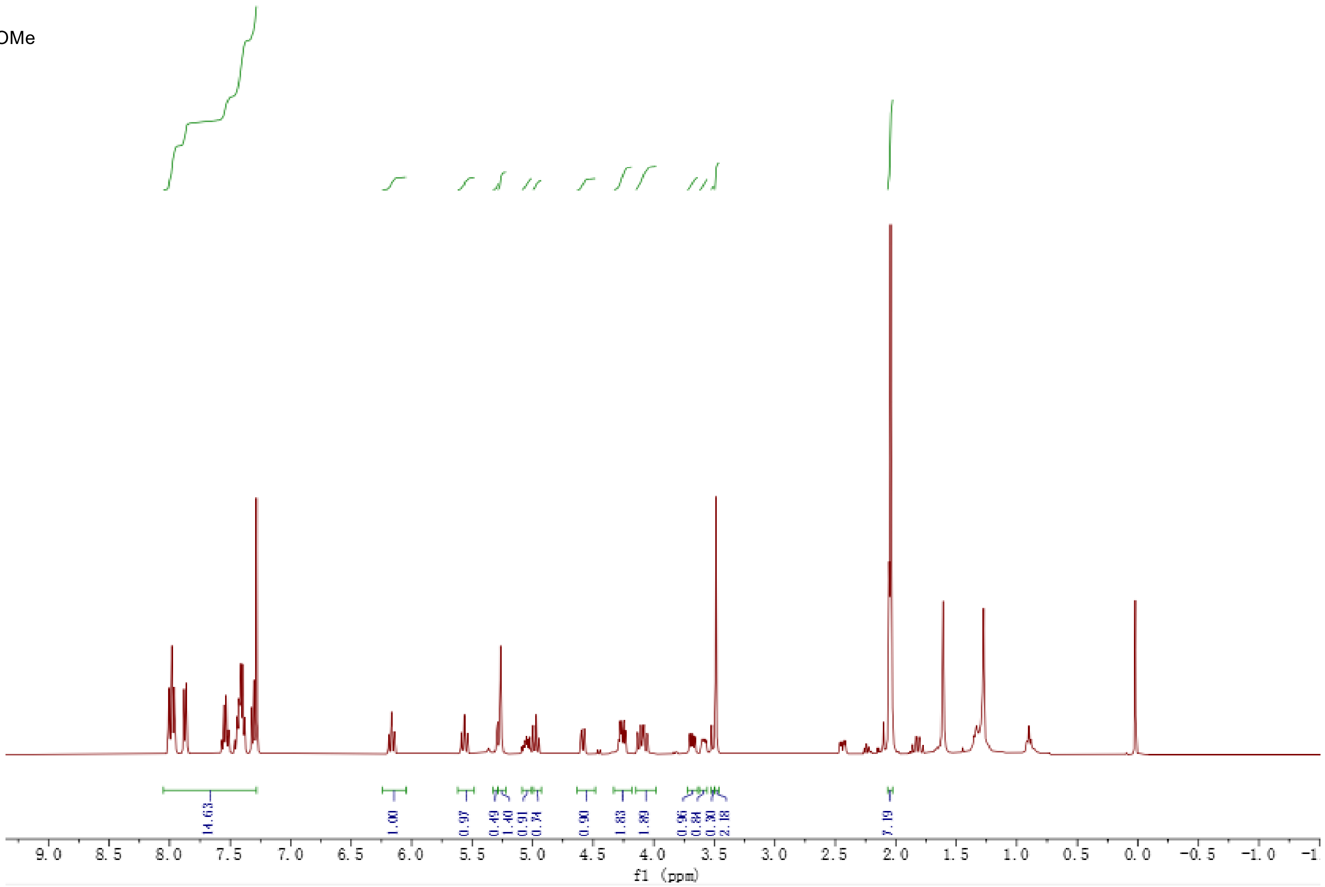

Figure S23. ^{1}H NMR spectrum (400 MHz) of **2aD** in $CDCl_3$

Methyl 2,3,4-tri-*O*-benzoyl-6-*O*-(3,4,6-tri-*O*-acetyl-2-deoxy-*β*-D-glucopyranoside)-*α*-D-glucopyranoside 2aD

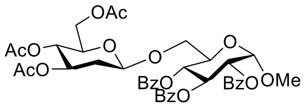

2aD

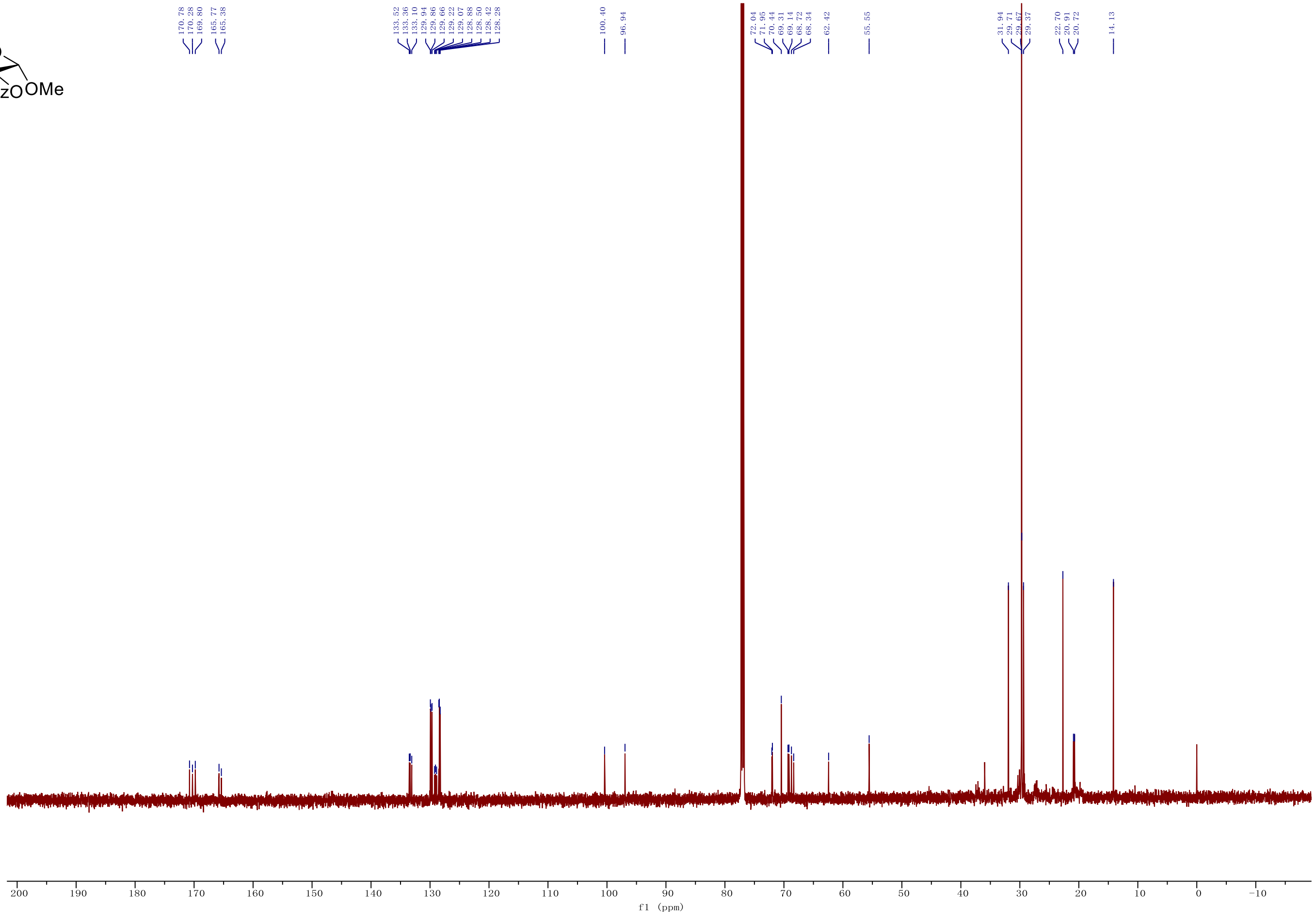

Figure S24. ^{13}C NMR spectrum (151 MHz) of **2aD** in $CDCl_3$

Methyl 2-*O*-benzoyl-4,6-*O*-benzylidene-3-*O*-(3,4,6-tri-*O*-acetyl-2-deoxy-*β*-D-glucopyranoside)-*α*-D-glucopyranoside 2eD

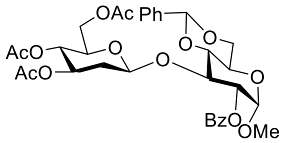

2eD

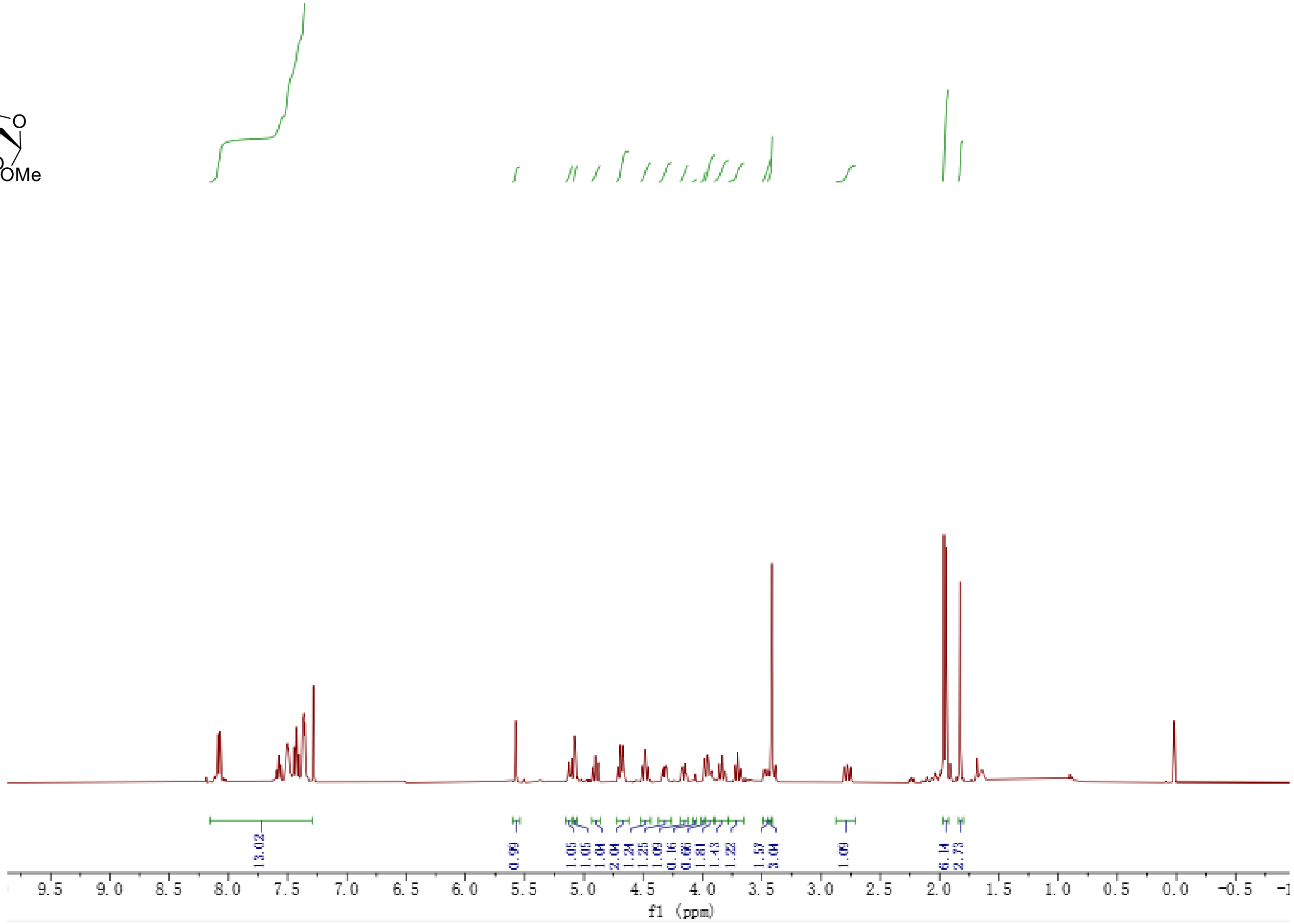

Figure S25. ^{1}H NMR spectrum (400 MHz) of **2eD** in $CDCl_3$

Methyl 2-*O*-benzoyl-4,6-*O*-benzylidene-3-*O*-(3,4,6-tri-*O*-acetyl-2-deoxy-*β*-D-glucopyranoside)-*α*-D-glucopyranoside 2eD

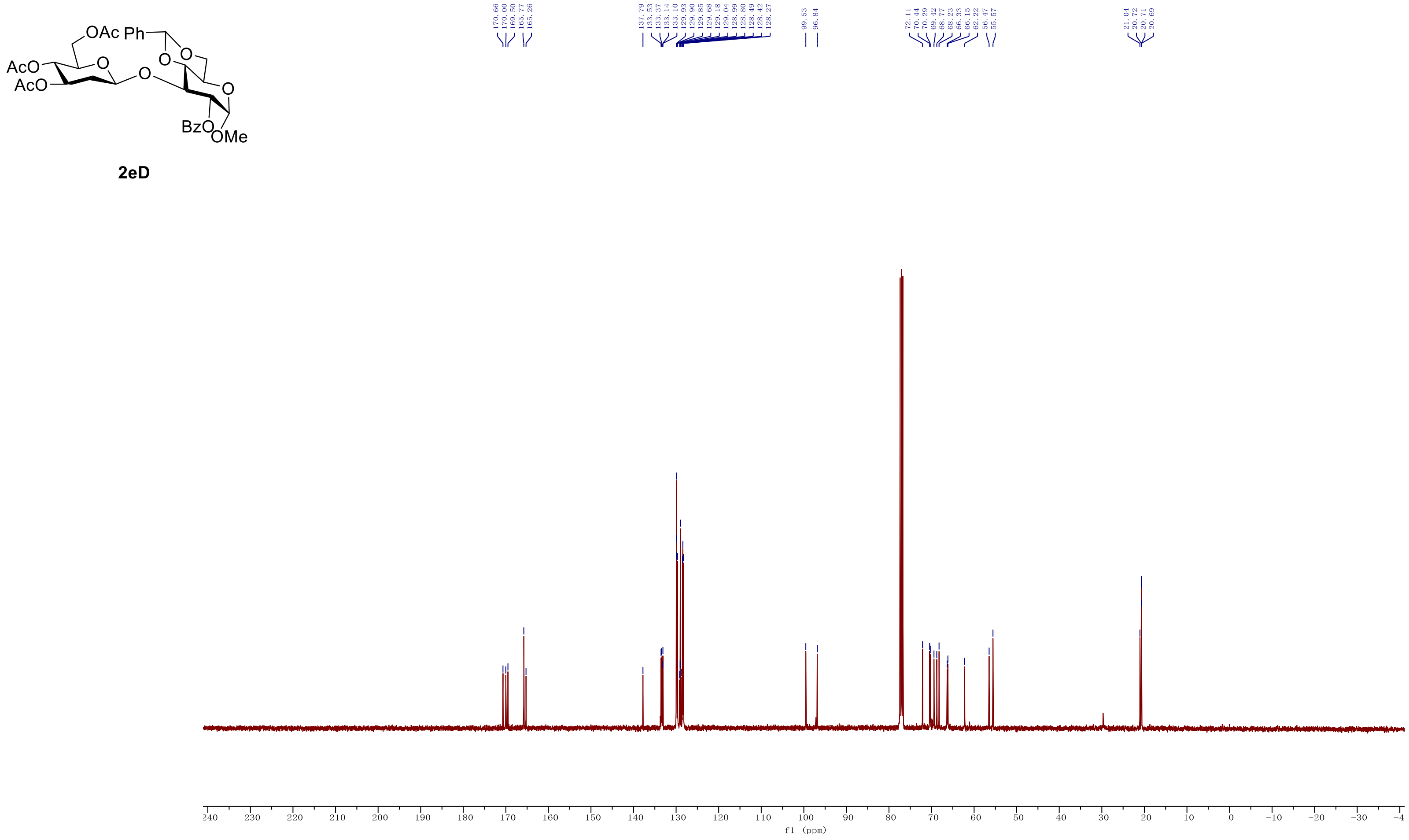

2eD

Figure S26. ^{13}C NMR spectrum (101 MHz) of **2eD** in $CDCl_3$

Methyl 3,4,6-tri-*O*-benzoyl-2-*O*-(3,4,6-tri-*O*-acetyl-*β*-D-glucopyranoside)-*β*-D-glucopyranoside 2fD

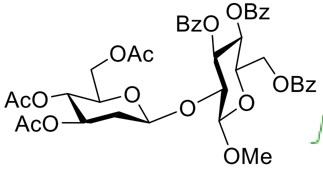

2fD

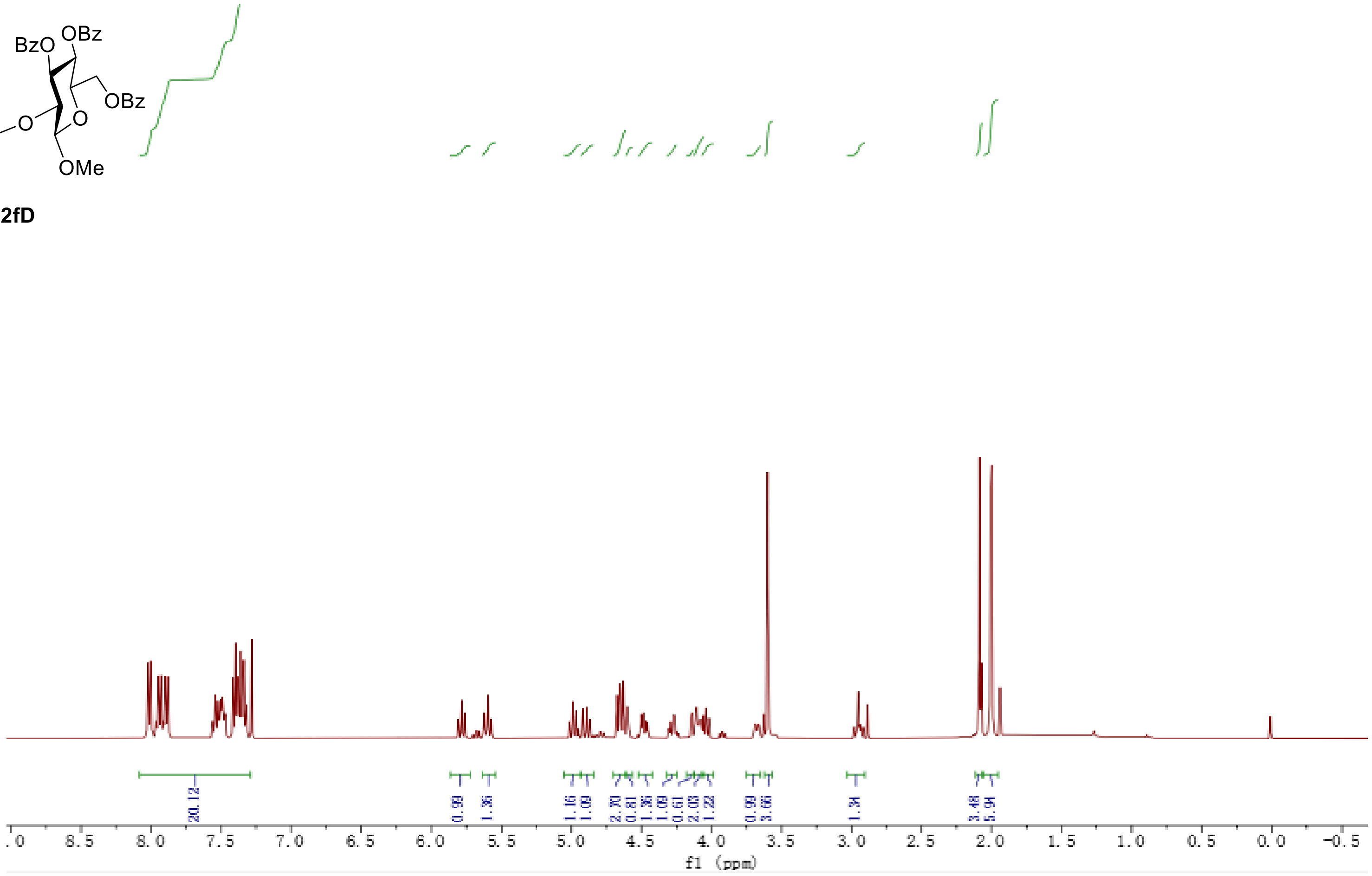

Figure S27. ^{1}H NMR spectrum (400 MHz) of **2fD** in $CDCl_3$

Methyl 3,4,6-tri-*O*-benzoyl-2-*O*-(3,4,6-tri-*O*-acetyl-*β*-D-glucopyranoside)-*β*-D-glucopyranoside 2fD

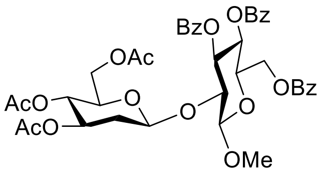

2fD

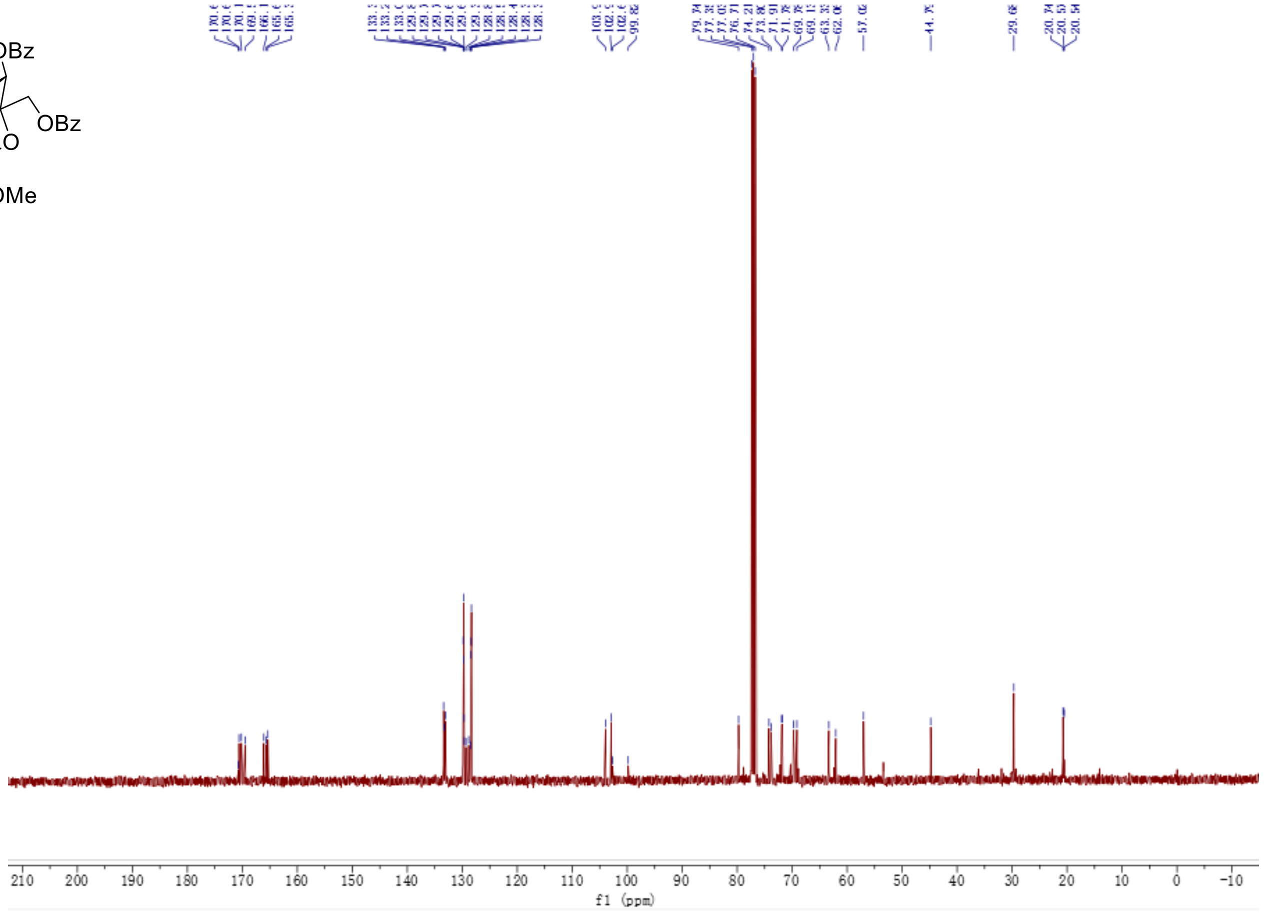

Figure S28. ^{13}C NMR spectrum (101 MHz) of **2fD** in $CDCl_3$
